# Evinacumab for Pediatric Patients With Homozygous Familial Hypercholesterolemia

**DOI:** 10.1161/CIRCULATIONAHA.123.065529

**Published:** 2023-10-20

**Authors:** Albert Wiegman, Susanne Greber-Platzer, Shazia Ali, M. Doortje Reijman, Eliot A Brinton, Min-Ji Charng, Shubha Srinivasan, Carissa Baker-Smith, Seth Baum, Julie A. Brothers, Jacob Hartz, Patrick M. Moriarty, Jeanne Mendell, Sébastien Bihorel, Poulabi Banerjee, Richard T. George, Boaz Hirshberg, Robert Pordy

**Affiliations:** 1 Department of Paediatrics, Amsterdam University Medical Centers, Location University of Amsterdam, The Netherlands (A.W., M.D.R.).; 2 Department of Pediatrics and Adolescent Medicine, Division of Pediatric Pulmonology, Allergology and Endocrinology, Medical University of Vienna, Austria (S.G.-P.).; 3 Regeneron Pharmaceuticals, Inc, Tarrytown, NY (S.A., J.M., S.B., P.B., R.T.G., B.H., R.P.).; 4 Utah Lipid Center, Salt Lake City (E.A.B.).; 5 Division of Cardiology, Department of Internal Medicine, Shin Kong Wu Ho-Su Memorial Hospital, Taipei, Taiwan (M.-J.C.).; 6 Institute of Endocrinology and Diabetes, Children’s Hospital at Westmead, Sydney, Australia (S.S.).; 7 University of Sydney, Australia (S.S.).; 8 Pediatric Preventive Cardiology Program, Nemours Cardiac Center, Nemours Children’s Hospital, Wilmington, DE (C.B.-S.).; 9 Flourish Research, Boca Raton, FL (S.B.).; 10 Division of Cardiology, Children’s Hospital of Philadelphia, PA (J.A.B.).; 11 Department of Cardiology, Boston Children’s Hospital, MA (J.H.).; 12 Department of Medicine, University of Kansas Medical Center, Kansas City (P.M.M.).

**Keywords:** angiopoietin-like protein 3, atherosclerosis, evinacumab, homozygous familial hypercholesterolemia, lipids, lipoprotein, pediatrics

## Abstract

**BACKGROUND:**

Homozygous familial hypercholesterolemia (HoFH) is a rare genetic disorder characterized by severely elevated low-density lipoprotein cholesterol (LDL-C) levels due to profoundly defective LDL receptor (LDLR) function. Given that severely elevated LDL-C starts in utero, atherosclerosis often presents during childhood or adolescence, creating a largely unmet need for aggressive LDLR-independent lipid-lowering therapies in young patients with HoFH. Here we present the first evaluation of the efficacy and safety of evinacumab, a novel LDLR-independent lipid-lowering therapy, in pediatric patients with HoFH from parts A and B of a 3-part study.

**METHODS:**

The phase 3, part B, open-label study treated 14 patients 5 to 11 years of age with genetically proven HoFH (true homozygotes and compound heterozygotes) with LDL-C >130 mg/dL, despite optimized lipid-lowering therapy (including LDLR-independent apheresis and lomitapide), with intravenous evinacumab 15 mg/kg every 4 weeks.

**RESULTS:**

Evinacumab treatment rapidly and durably (through week 24) decreased LDL-C with profound reduction in the first week, with a mean (SE) LDL-C reduction of −48.3% (10.4%) from baseline to week 24. ApoB (mean [SE], –41.3% [9.0%]), non–high-density lipoprotein cholesterol (–48.9% [9.8%]), and total cholesterol (–49.1% [8.1%]) were similarly decreased. Treatment-emergent adverse events were reported in 10 (71.4%) patients; however, only 2 (14.3%) reported events that were considered to be treatment-related (nausea and abdominal pain). One serious treatment-emergent adverse event of tonsillitis occurred (n=1), but this was not considered treatment-related.

**CONCLUSIONS:**

Evinacumab constitutes a new treatment for pediatric patients with HoFH and inadequately controlled LDL-C despite optimized lipid-lowering therapy, lowering LDL-C levels by nearly half in these extremely high-risk and difficult-to-treat individuals.

**REGISTRATION:**

URL: https://www.clinicaltrials.gov; Unique identifier: NCT04233918.

**Clinical Perspective**

**What Is New?**

- Until now, limited treatment options were available to help pediatric patients with homozygous familial hypercholesterolemia. Recently, evinacumab, an angiopoietin-like 3 inhibitor, was approved by the US Food and Drug Administration as an adjunct to other lipid-lowering therapies to treat patients 5 to 11 years of age with homozygous familial hypercholesterolemia.

**What Are the Clinical Implications?**

- This first Food and Drug Administration–approved treatment provides an effective treatment for these very young patients with homozygous familial hypercholesterolemia, and potentially aids achievement of recommended low-density lipoprotein cholesterol levels much earlier in the course of this rare disease.

**Editorial, see p 363**

Familial hypercholesterolemia is a monogenic semidominant hypercholesterolemia caused by pathogenic DNA variants in the low-density lipoprotein receptor (*LDLR*) gene or other genes related to LDLR function, including apolipoprotein B (*APOB*), proprotein convertase subtilisin/kexin type 9 (*PCSK9*), and LDLR adaptor protein 1 (*LDLRAP1*).^1–3^ Homozygous familial hypercholesterolemia (HoFH) has a prevalence of approximately 1 in 250 000 to 360 000 individuals worldwide and is characterized by severe hypercholesterolemia, with low-density lipoprotein cholesterol (LDL-C) levels generally >400 mg/dL (>10 mmol/L),^2^ starting in utero.^4,5^ HoFH includes patients with LDLR-defective (some residual activity; ranging between 2% and 15%), and with LDLR-negative (no activity [<2%]) pathogenic variants.^2,6–11^ Elevated LDL-C levels lead to a roughly 20-fold or more increased risk of developing premature atherosclerotic cardiovascular disease complications, including coronary arterial atherosclerosis as well as valvular and supravalvular aortic stenosis, compromising the coronary ostia (that results from accumulation of lipids in the aortic root).^2^ This pathology can lead to early myocardial infarction and other atherosclerotic cardiovascular disease events, resulting in disability or death during childhood and adolescence.^12–14^

The magnitude of LDL-C elevations in HoFH is primarily dependent on the gene involved, with pathogenic variants in *LDLR* associated with more severe phenotypes compared with pathogenic variants in *APOB*, *PCSCK9*, and *LDLRAP1*, and more severe outcomes associated with LDLR-negative variants compared with LDLR-defective variants.^2^

In the United States, approved standard-of-care lipid-lowering therapies (LLTs) used to reduce LDL-C in adult and pediatric patients with HoFH include statins, ezetimibe, PCSK9 inhibitors, and lipoprotein apheresis.^2,15–19^ LLTs solely approved for the treatment of adult patients with HoFH include bempedoic acid and lomitapide.^2,19–21^ Because the 4 main classes of LLTs (ie, statins, ezetimibe, bempedoic acid, and PCSK9 inhibitors) are LDLR-dependent, they have minimal efficacy in patients with HoFH and almost none in those with null/null variants.^19,22–28^ Consequently, despite the use of multiple LLTs, the majority of patients with HoFH fail to reach their guideline-recommended LDL-C targets.^29^ Lomitapide and lipoprotein apheresis are advanced LLTs that act independently of LDLR function.^30,31^ However, lomitapide can cause hepatic fat accumulation, which necessitates the need for a low-fat intake and dose titration to control gastrointestinal symptoms, and therefore is not available for the pediatric population.^2,21^ Although lipoprotein apheresis can be used in patients with HoFH from about 3 years of age,^2^ each treatment is time-consuming and lengthy, availability is limited, and it is a relatively invasive therapy that can adversely affect patients’ quality of life.^2,32^ Liver transplantation is an important but uncommon LDLR-independent curative treatment option that reverses the hepatic cholesterol metabolism abnormalities in patients with HoFH.^33^ However, liver transplantation is limited by scarcity of organ donors, and is associated with disadvantages such as high risk of post-transplantation surgical complications and mortality, and the necessity for life-long immunosuppressive therapy.^2,33^ Given the limited treatment options, there remains a large unmet need for LLTs that are both effective and well-tolerated in pediatric patients with HoFH.

Recently, angiopoietin-like 3 (ANGPTL3) has emerged as a target for the treatment of elevated LDL-C levels. Individuals with familial combined hypolipidemia with biallelic loss-of-function pathogenic variants in the *ANGPTL3* gene have extremely low LDL-C levels and a substantially reduced risk of atherosclerotic cardiovascular disease.^34^ Inhibition of ANGPTL3 reduces LDL-C independent of the LDLR, possibly by promoting removal of very low-density lipoprotein (VLDL) before it can be converted to LDL (low-density lipoprotein).^35^

Evinacumab is a fully human monoclonal antibody that selectively binds to and inhibits ANGPTL3.^35^ In the phase 3 ELIPSE HoFH trial (Evinacumab Lipid Studies in Patients with Homozygous Familial Hypercholesterolemia), evinacumab, when administered with other LLTs, lowered LDL-C by approximately 50% in adult and adolescent patients ≥12 years of age with HoFH, and was generally well-tolerated.^36,37^ Furthermore, a recent study of 2 adolescent patients (12 and 16 years of age) with HoFH and null/null *LDLR* variants who participated in the ELIPSE HoFH trial demonstrated that plaque volumes were reduced by 76% and 85% after 6 months of evinacumab treatment, as assessed by coronary computed tomography angiography.^38^ On the basis of the ELIPSE HoFH trial, evinacumab was initially approved by the US Food and Drug Administration in February 2021 and by the European Medicines Agency in June 2021, as an adjunct to other LDL-C–lowering therapies for the treatment of patients ≥12 years of age with HoFH.^36,37,39^ In March 2023, the Food and Drug Administration approval of evinacumab was extended to treat patients 5 to 11 years age of with HoFH, such as those who participated in this study.^36^

This article reports the first study of the efficacy and safety of evinacumab in pediatric patients 5 to 11 years of age with HoFH.

## METHODS

This series of open-label, 3-part studies was conducted at 10 sites across Australia, Austria, The Netherlands, Taiwan, Ukraine, and the United States, and was designed to assess the efficacy and safety of evinacumab in pediatric patients with HoFH (REGISTRATION: URL: https://www.clinicaltrials.gov; Unique identifier: NCT04233918). All patients were counseled to continue standard dietary measures for LDL-C–lowering on all visits throughout all parts of the trial.

The trial was conducted in accordance with the ethical principles of the Declaration of Helsinki and was consistent with the International Conference on Harmonization Good Clinical Practice Guidelines. Approvals were obtained from the health authorities or ethics committees (including institutional review boards) to enable the initiation of study sites for this study, as allowed by local laws and regulations. Informed written consent was obtained from each patient and their parents or legal guardians before participation in the study. All patients were offered the study drug after completion of the trial.

### Data Availability

Qualified researchers may request access to study documents (including the clinical study report, study protocol with any amendments, blank case report form, and statistical analysis plan) that support the methods and findings reported in this article. Individual anonymized participant data will be considered for sharing after the product and indication have been approved by major health authorities (eg, Food and Drug Administration, European Medicines Agency, Pharmaceuticals and Medical Devices Agency, etc) if there is legal authority to share the data and there is not a reasonable likelihood of participant reidentification. Submit requests to https://vivli.org/.

### Study Design and Treatment

This was a 3-part, open-label study (Figure S1). All participants were pediatric patients 5 to 11 years of age with HoFH.

Part A was a phase 1b, single-dose, open-label, 16-week study that assessed the safety, pharmacokinetics, and pharmacodynamics of evinacumab in 6 patients. After a ≤8-week run-in period, there was a 1- to 2-week screening period, followed by a 16-week treatment and observation period during which patients received a single dose of evinacumab 15 mg/kg administered intravenously. Per protocol, patients in part A receiving lipoprotein apheresis temporarily discontinued apheresis from the baseline visit to 4 weeks after receiving the single dose of evinacumab. In addition, the duration between the previous apheresis treatment and the baseline visit had to be at least either 14 or 7 days, depending on the frequency of the apheresis. Patients were allowed to resume their apheresis schedule after completing all week 4 assessments. Part A established safety and tolerability, and determined that evinacumab pharmacokinetics in pediatric patients was comparable to adults at this dose, therefore allowing continuation of the dose in parts B and C.

Part B was a phase 3, single-arm, 24-week open-label study that assessed the efficacy, safety, and pharmacokinetics of evinacumab in 14 patients who had not participated in part A. Part B consisted of a ≤8-week run-in, 1 to 2 weeks of screening, and 24 weeks of treatment. For patients on lipoprotein apheresis, evinacumab was administered within 1 day of completing apheresis.

Part C is an ongoing, phase 3, 48-week open-label extension study with a 24-week follow-up period, designed to assess the long-term safety and efficacy of evinacumab, which includes all 20 patients from both parts A and B.

During the conduct of the study, adherence to lipid-modifying therapy was assessed by the investigator at each visit during parts A, B, and C and recorded in the study database.

### Eligibility Criteria

Patients 5 to 11 years of age with LDL-C >130 mg/dL despite aggressive LLT at the screening visit, and with a diagnosis of functional HoFH by genetic or clinical criteria, were eligible for the study. The full list of patient eligibility criteria is provided in the Supplemental Material.

### Study Objectives and End Points

The objectives of part A were to assess the pharmacokinetics parameters for total evinacumab (either 1 or 2 unoccupied binding sites as well as both binding sites occupied) and total ANGPLT3 (either 1 or 2 unoccupied binding sites as well as both binding sites occupied), and the safety and tolerability of evinacumab in pediatric patients with HoFH.

The primary efficacy objective for part B was to quantify the mean percent change in LDL-C with evinacumab from baseline to week 24 (intention-to-treat [ITT] population). The baseline LDL-C value was defined as the last LDL-C value obtained before the first dose of evinacumab in part B.

Secondary objectives for part B included evaluation of the effect of evinacumab on lipid and lipoprotein levels, including apoB, non–high-density lipoprotein cholesterol (HDL-C), total cholesterol, and lipoprotein (a) (Lp[a]) on the basis of the mean percent change in these measurements from baseline to week 24 (ITT population). The safety and tolerability of evinacumab were also assessed at week 24 on the basis of the incidence of treatment-emergent adverse events (TEAEs) and safety variables over time. Pharmacokinetics, including total evinacumab and total ANGPTL3 concentrations, and immunogenicity, including antidrug antibodies (ADAs) and neutralizing antibodies, were also assessed.

A list of all primary and secondary end points for part B are provided in the Supplemental Material.

### Lipid Measurements

All blood samples were collected before administering evinacumab on a given day. In addition, for patients receiving lipoprotein apheresis, blood samples were taken before apheresis treatment for that day. Blood samples were collected (after an overnight fast, always requested, done whenever possible) for the assessment of lipid profiles, including LDL-C, apoB, HDL-C, non–HDL-C, total cholesterol, Lp(a), and triglycerides. LDL-C was calculated using the Friedewald equation^40^ when triglycerides were >400 mg/dL (4.52 mmol/L). When calculated, LDL-C was <25 mg/dL (0.65 mmol/L), and LDL-C was determined by beta quantification.^41^

### Pharmacokinetics and Immunogenicity Assessments and Bioanalytical Methods

Samples for evinacumab and total ANGPTL3 concentrations were collected at baseline and on days 8, 15, 22, 29, 43, 57, 71, 85, 113, and 169. These samples were collected before the dose and at the end of the intravenous infusion on days when evinacumab was administered. Serum samples were analyzed for total evinacumab and total ANGPLT3 using validated ELISAs with a lower limit of quantitation of 0.078 mg/L and 0.0195 mg/L, respectively. ADA samples were collected at baseline, and both at the end of treatment (day 113) and the end of the study (day 169). ADAs were assessed in serum samples using a validated, titer-based bridging immunoassay. Samples positive in the ADA assay were analyzed in the neutralizing antibody assay. In case of a suspected serious adverse event such as hypersensitivity or an anaphylactic reaction, additional samples were collected at or near the time of the event using a validated competitive ligand-binding assay.

For the pharmacokinetics analysis, the observed concentrations of evinacumab in serum for patients in part A were compared with individual population predictions obtained from a previously developed 2‐compartment population pharmacokinetics model with combined linear and saturable (Michaelis-Menten) elimination and first‐order absorption.^42^ Data for the population pharmacokinetics model developed for the adult population were pooled from 6 evinacumab clinical studies and comprised healthy volunteers and patients with HoFH. The population pharmacokinetics model contained both fixed effects (parameter estimates) and random effects (variability). Baseline ANGPTL3 and body weight were important determinants or covariates for the pharmacokinetics parameters in the model. The population pharmacokinetics model was used to predict concentrations for the individual pediatric patients using their baseline ANGPTL3 and body weight values. Concentration-time profiles were plotted for the 2.5th, 50th, and 97.5th percentiles of the predicted data and overlaid with the observed data.

### Subgroup Analyses

To evaluate possible heterogeneity of the treatment effect across various subgroups, the percent change in LDL-C from baseline to week 24 was assessed by sex, younger (≥5 to <10 years) or older (≥10 to <12 years) age, race, ethnicity, baseline apheresis status (yes or no), and LDLR activity (negative/negative [LDLR activity <2%], defective/negative [LDLR activity >1%], or defective/defective [>2%]).^43^

### Statistical Analysis

No formal statistical testing or calculation of desired sample size was conducted before the study. For the purpose of estimating exposures, the concentrations of total evinacumab over time as well as the selected pharmacokinetics parameters for part A and part B were summarized descriptively as geometric means. The pharmacokinetics analysis population included all patients who received any study drug and who had at least one nonmissing result for evinacumab concentration after the first dose of the study drug. The total target analysis population included all patients who received any study drug and who had at least one nonmissing result for total ANGPTL3 concentration after the first dose of the study drug. Similarly, the ADA analysis population included all patients who received any study drug and who had at least 1 nonmissing ADA result after the first dose of the study drug.

The efficacy end points in part B were assessed in the ITT population (defined as all patients who received ≥1 dose of evinacumab). For the primary end point, the percent change from baseline in calculated LDL-C at week 24 was analyzed in the ITT population using a pattern-mixture model approach. Baseline LDL-C values were incorporated into the end point, as the percent change from baseline in calculated LDL-C was analyzed. Two different imputation strategies were performed for missing LDL-C values, one for LDL-C values missing during the on-treatment period and one for missing LDL-C values after the on-treatment period. Missing LDL-C values during the on-treatment period were imputed with the assumption that LDL-C values were “missing at random” and imputed on the basis of other observed measurements in the on-treatment period. Because the pattern of missing data was expected to be non-monotone, the Monte Carlo Markov chain method with the IMPUTE=MONOTONE option in PROC MI (Statistical Analysis Software version 9.4, SAS Institute, Cary, NC) was used to create an imputed data set with a monotone missing pattern (ie, with the on-treatment missing LDL-C values imputed). The Monte Carlo Markov chain method used a single chain to produce 100 imputations, and by default, 200 burn-in iterations were completed before the first imputation and 100 iterations between imputations. Missing LDL-C values during the post-treatment period were imputed assuming patients who stopped taking their study treatment no longer benefited from it after discontinuation and thus tended to have LDL-C values returning to baseline. For each patient, missing post-treatment LDL-C values were imputed using a random draw from a normal distribution, with a mean equal to the patient’s own baseline value and variance equal to the conditional variance at the specific time point, given the baseline value. Although there was a prespecified procedure for imputation of missing values, 100% of LDL-C data was available for analysis of the primary efficacy end point at week 24.

The 100 complete data sets of observed and imputed calculated LDL-C data at week 24 were analyzed using the SAS MEANS procedure. The SAS MIANALYZE procedure was used to generate valid statistical inferences by combining results from the 100 analyses using Rubin’s formulae. The model provided the combined estimate for the mean at week 24 with the SE and 95% CI. The robustness of this statistical method was assessed through sensitivity analyses, including an ITT analysis and an on-treatment analysis of the percent change in LDL-C from baseline to week 24 using a mixed-effects model with a repeated-measures approach. All postbaseline data available within the week 1 to 24 analysis windows were used, regardless of adherence to treatment and subsequent therapies, and missing data were accounted for by the mixed-effects model with repeated measures. The model included the fixed categorical effect of time point (weeks 1, 2, 4, 8, 12, 16, 20, and 24) and the continuous fixed covariate of baseline LDL-C value.

For the secondary efficacy end points, descriptive summaries and analyses were performed in the ITT population, using LDL-C values obtained within the week 24 efficacy analysis window, regardless of adherence to study treatment and subsequent therapies (ITT estimand). The subgroup analyses were performed using the same ITT estimand and pattern-mixture model approach for missing data as were used for the primary end point. Testing for control of multiplicity was not applicable to this analysis.

The safety assessments were conducted in the safety analysis set, which included the same patients as the efficacy analysis set. Further details of statistical analyses are provided in the Supplemental Material.

## RESULTS

### Part A

**Figure 1.**
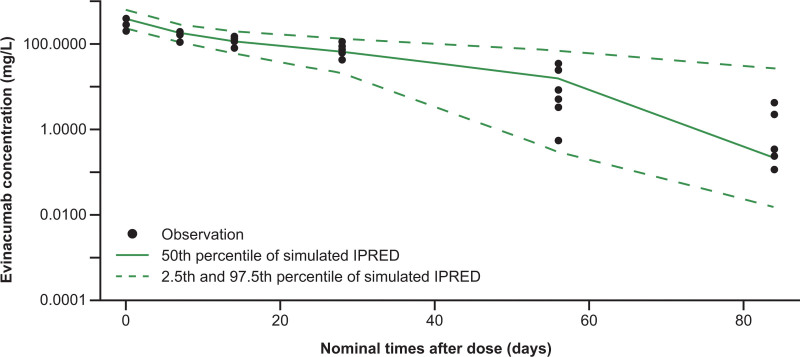
**Evinacumab serum concentration over time after a single dose of evinacumab in part A.** Six patients received a single dose of evinacumab (15 mg/kg intravenously). The observed (●) evinacumab serum concentrations were consistent with the model-based predictions (individual population predictions [IPRED]).

All 6 enrolled patients completed part A of the study. The mean (SD) age of the patients was 8.8 (1.7) years, 4 were female, and all were White, with a mean (SD) body mass index of 17.3 (2.8) kg/m^2^. The observed evinacumab serum concentrations were within the pharmacokinetics model–based prediction intervals (Figure 1), confirming the regimen of 15 mg/kg every 4 weeks because exposure was comparable in both pediatric and adult patients who participated in the phase 3 studies. In part A, a single dose of evinacumab led to a mean (SD) incremental LDL-C reduction of 41.6% (18.3%) after 8 weeks (Figure S2).

### Part B

#### Patient Disposition and Baseline Characteristics

Of the 16 screened pediatric patients in part B, 14 were enrolled. All enrolled patients received evinacumab every 4 weeks until week 24 (until the end of the study) and were included in both the efficacy/ITT and the safety analysis sets (Figure S3).

The demographic and baseline characteristics of patients from part B are presented in Table 1. The median (minimum:maximum) age of patients was 9.5 (5:11) years, and 57.1% were girls. Baseline lipid parameters are summarized in Table 2. At baseline, 7 of 14 patients (50%) were receiving lipoprotein apheresis; all 14 patients were also receiving other nonstatin LLTs (Table 1). *LDLR* genotyping showed that most patients were compound heterozygous (71.4%) and belonged to the defective/defective, the defective/negative, as well as negative/negative LDLR variant categories (Table S1).

**Table 1.**
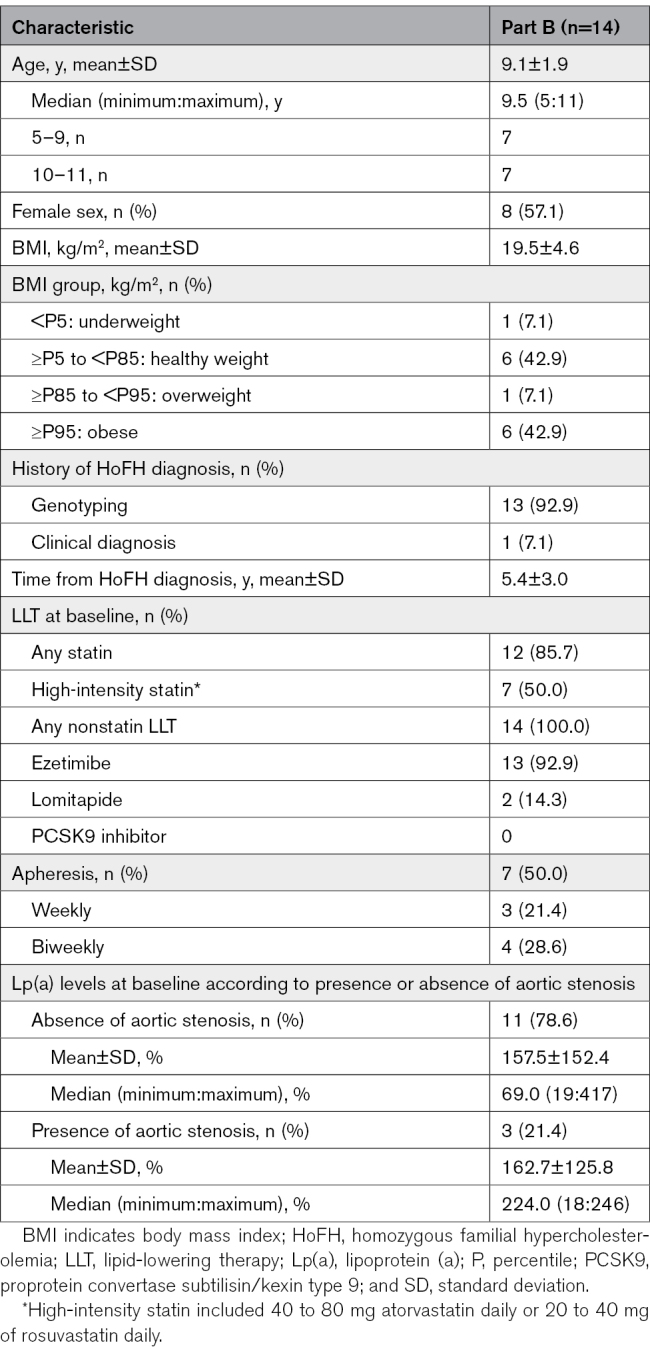
Baseline Demographic and Clinical Characteristics of Patients From Part B

**Table 2.**
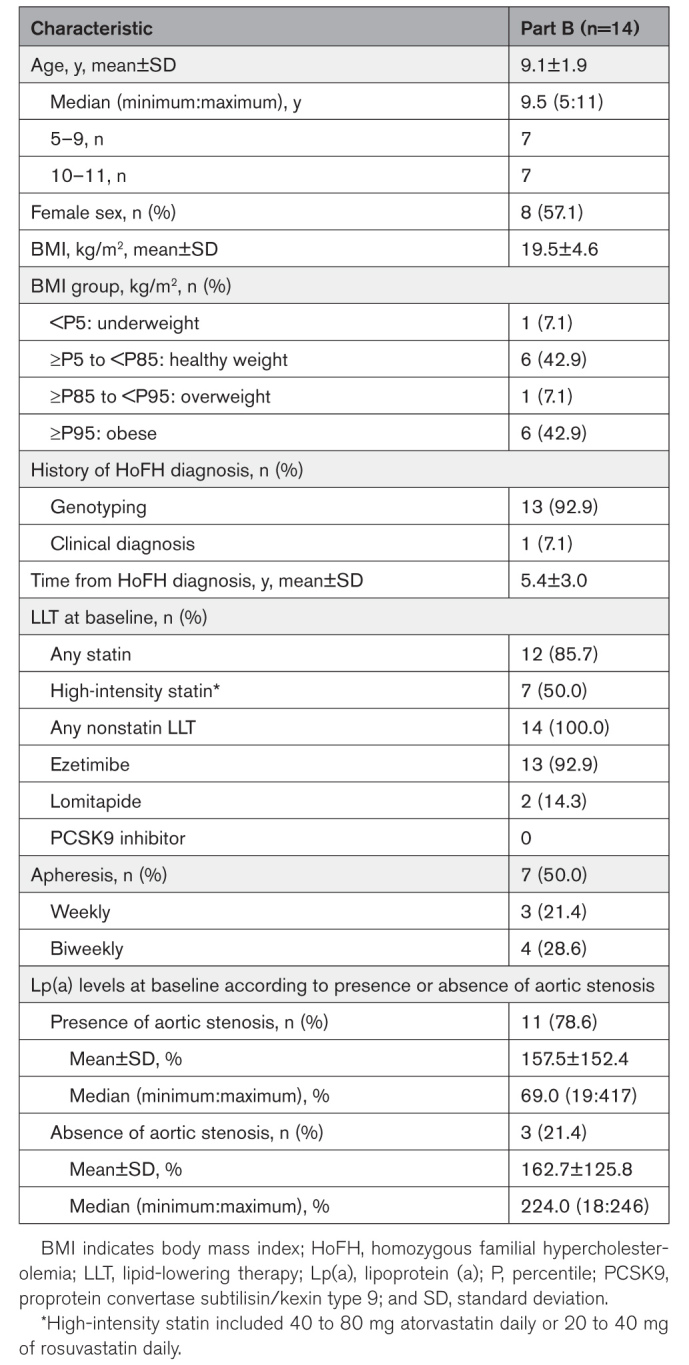
Baseline Lipid Parameters at Study Entry in Part B (ITT Population)

#### Efficacy Outcomes

**Figure 2.**
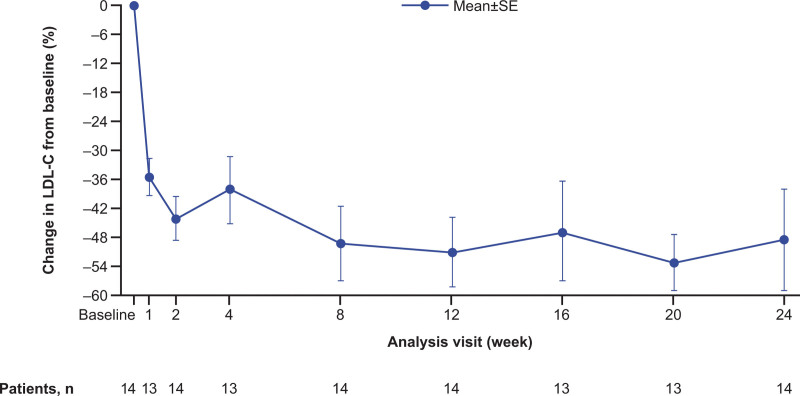
**Percent change in calculated LDL-C from baseline to week 24 (intention-to-treat population).** Raw data are shown, as there were no missing data at the primary efficacy time point (24 weeks). LDL-C was calculated using the Friedewald equation except when triglycerides were >400 mg/dL (4.52 mmol/L) or calculated LDL-C was <25 mg/dL (0.65 mmol/L), in which case LDL-C was determined by beta quantification. LDL-C indicates low-density lipoprotein cholesterol; and SE, standard error.

Mean (SE) LDL-C at baseline was 263.7 (24.3) mg/dL. For the primary end point, the mean (SE) percent change in LDL-C from baseline was –48.3% (10.4%) at week 24 (Table 3). The mean (SE) absolute change in LDL-C from baseline at week 24 was –131.9 (30.0) mg/dL. LDL-C reductions from baseline occurred rapidly and were consistent from week 1 to week 24 (Figure 2). Although the overall mean percent reduction in LDL-C at week 24 in all patients was –48.3% (n=14), in a post hoc analysis, this metric was −62.8% if one patient with documented noncompliance to concomitant medications and one patient suspected of noncompliance (Figure S4) are excluded. The mean percent change in LDL-C from baseline at week 24 for each individual patient from part B is shown in Figure S5.

**Table 3.**
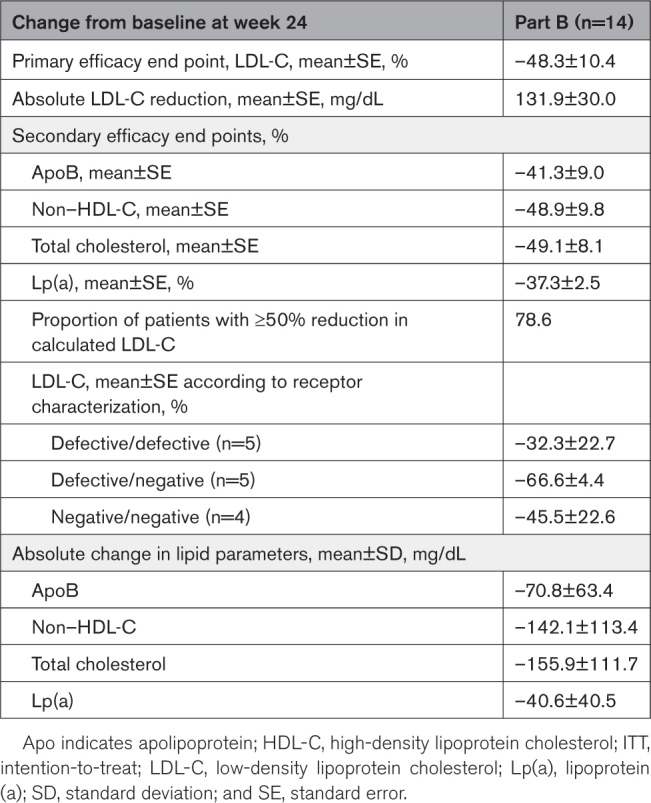
Summary of Primary, Secondary, and Other End Points at Week 24 From Part B (ITT Population)

Subgroup analyses showed that the LDL-C reduction achieved with evinacumab was similar regardless of sex, race, ethnicity, baseline apheresis status, and imputed LDLR activity (Figure S6). The mean (95% CI) percent change from baseline in LDL-C at week 24 was –65.7% (–72.3% to –59.2%) and –30.9% (–68.1% to 6.3%) for 7 patients ≥5 to <10 years of age and 7 patients ≥10 to <12 years of age, respectively. The lesser reduction from baseline in LDL-C for the patients ≥10 to <12 years of age appeared to be largely due to the documented nonadherence to nonstudy LLTs of one patient who had an associated increase in LDL-C at week 24.

Further post hoc analyses showed that LDL-C reduction at week 24 with evinacumab was similar whether baseline LDL-C values were above or below the median values, and also regardless of lomitapide treatment (Table S2). In addition, the percent change from baseline in Lp(a) at week 24 was similar regardless of baseline apheresis status (Table S3). According to receptor characterization, the mean (SE [95% CI]) percent changes from baseline in LDL-C at week 24 with evinacumab in patients in the defective/defective (n=5), defective/negative (n=5), and negative/negative (n=4) LDLR categories were –32.3% (22.7 [95% CI, –76.8% to 12.3%]), –66.6% (4.4 [95% CI, –75.1% to –58.0%]), and –45.5% (22.6 [95% CI, –89.9% to –1.2%]), respectively (Table 3).

In addition, mean percent changes from baseline at week 24 with evinacumab were observed for apoB (–41.3%), non–HDL-C (–48.9%), total cholesterol (–49.1%), and Lp(a) (–37.3%) (Table 3, Figure S7). Furthermore, most patients (78.6%) treated with evinacumab showed a ≥50% reduction in LDL-C at week 24 from baseline.

#### Safety Outcomes

During part B, at least one TEAE occurred in 10 (71.4%) patients with HoFH treated with evinacumab (Table 4). Serious TEAEs during the treatment period occurred in one patient, who was admitted to the hospital with fever and leukocytosis with suspected indwelling vascular catheter infection. Tonsillitis was subsequently identified and treated, and the patient was discharged home after showing marked improvement; the event was not considered treatment-related. Severe TEAEs occurred in 3 (21.4%) patients, including aortic stenosis, unilateral deafness, and tonsillitis; none were considered treatment-related. Common TEAEs (occurring in ≥10% of patients) included oropharyngeal pain (3 [21.4%] patients), followed by upper abdominal pain, diarrhea, nausea, vomiting, headache, and nasopharyngitis (2 [14.3%] patients each). One patient experienced mild allergic events (rash and contact dermatitis). No patients experienced adverse events leading to treatment discontinuation, and no deaths were reported during the study period.

**Table 4.**
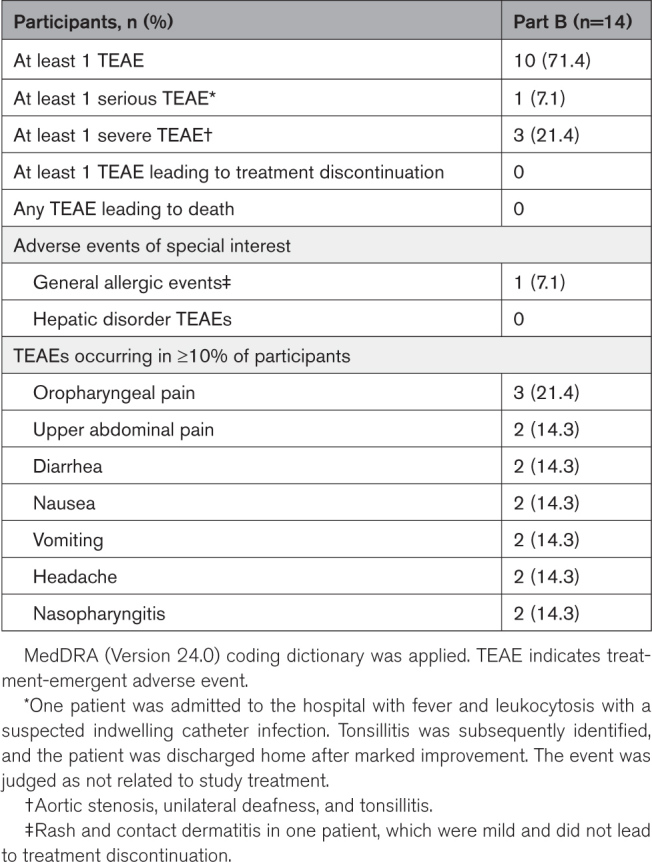
Overview of TEAEs in Part B (Safety Set)

#### Pharmacokinetics and Immunogenicity Outcomes

**Figure 3.**
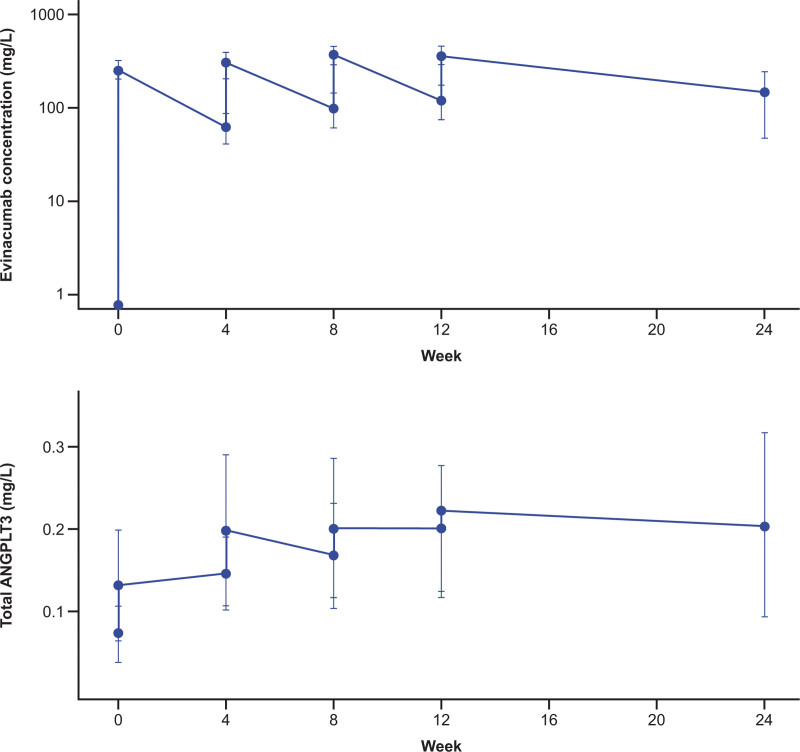
**Mean (±SD) predose and postdose concentrations of total evinacumab and ANGPTL3 vs time in part B.** Log-scaled evinacumab concentrations below the lower limit of quantification (LLOQ) (0.078 mg/L) were set to 1/2 LLOQ before computation of means (±SD). ANGPTL3 indicates angiopoietin-like 3; and SD, standard deviation.

The pharmacokinetics profiles of evinacumab demonstrated that all patients were compliant with evinacumab weekly treatment and that steady-state concentrations were attained after 8 to 12 weeks of treatment (Figure 3). Lipoprotein apheresis had a modest effect on evinacumab concentrations, with a 10% to 25% reduction. Evinacumab accumulated approximately 2-fold, regardless of apheresis treatment.

Total ANGPTL3 concentrations, ANGPLT3 alone, and ANGPLT3 complexed with evinacumab increased with evinacumab treatment and reached a plateau by approximately 12 weeks, indicating that target saturation was attained (Figure 3).

One patient in part A and another in part B had detectable pre-existing ADAs against evinacumab at the start of treatment. Neither of these 2 patients with ADAs detected before dose had previous exposure to evinacumab. It is important to note that neither patient gave positive results in a neutralizing antibody assay.

In addition, 2 patients had ADAs during part B. One patient had ADAs detectable before the dose and subsequently tested negative for ADAs. The other patient did not have ADA detectable before the dose but developed ADAs during treatment, which were therefore considered treatment-emergent ADAs. Neutralizing antibodies were not detected for either patient. The clinical efficacy of evinacumab (only assessed in part B) was not affected by the presence of these ADAs. For the patient with pre-existing ADAs and the patient with treatment-emergent ADAs, the LDL-C reduction was consistent across the study and with the population mean. The week 24 reductions from baseline were 52.1% and 77.7% for the patients with pre-existing and treatment-emergent ADAs, respectively, which was comparable to the mean LDL-C reduction of 48.3% among all patients in part B. Of note, both of these patients had low titers (ie, 50) of the ADAs detected.

## DISCUSSION

Currently, treatment options for children with HoFH 5 to 11 years of age are limited, because standard (LDLR-dependent) LLTs are insufficiently effective in HoFH (whether formally approved or not). In this study of pediatric patients 5 to 11 years of age with HoFH, evinacumab rapidly (within 1 week) and durably lowered LDL-C, with considerable reductions of 48% by week 24 (achieving the primary end point), similar to the 49% decrease seen at week 24 in a phase 3 study among adolescent and adult patients with HoFH.^37^ The 15-mg/kg dose of evinacumab resulted in concentrations that attained steady state by approximately 8 to 12 weeks, at which point saturation of ANGPTL3 was achieved. This decrease was incremental to the LDL-C-lowering effects of the other LDLR-independent treatments, lipoprotein apheresis, and lomitapide, which were generally continued, along with an LDL-C–lowering diet through the study (according to self-reporting).

At week 24, treatment with evinacumab was associated with an absolute mean reduction in LDL-C of –131.9 mg/dL (from a baseline of 263.7 mg/dL). The LDL-C was lowered by >50% in 11 of 14 patients (78.6%), with a similar effect regardless of apheresis use. It is important to note that effective LDL-C–lowering was reported across all subgroups, including those with LDLR-negative status. In particular, the finding of no attenuation of LDL-C–lowering efficacy in patients with the most severe HoFH is both reassuring and clinically relevant. Furthermore, reductions in apoB, non–HDL-C, and total cholesterol were also seen and were roughly comparable to those of LDL-C.

At week 24, Lp(a) was also reduced by a mean (SE) of 37% (2.5%), in contrast with the previous large pivotal study of evinacumab in adolescent and adult patients with HoFH, in which the mean (SE) reduction in Lp(a) from baseline at week 24 was 5.5% (4.0%).^37^ The reason for this difference is unclear, but it might in part be due to differences in the genetic makeup of younger patients with HoFH, higher mean baseline Lp(a) levels, or possibly the lack of use of PCSK9 inhibitors in the current pediatric study. Although Lp(a) levels are frequently elevated in HoFH,^44^ it has not yet been proven that lowering of Lp(a) levels may improve cardiovascular outcomes.^45^

Most patients with HoFH do not achieve the guideline-recommended LDL-C levels of <70 mg/dL.^2^ Because standard LLTs such as statins and PCSK9 inhibitors reduce LDL-C by upregulating LDLR expression, they have limited efficacy in HoFH. Lomitapide, which reduces LDL-C independently of the LDLR, is an established treatment for HoFH, but its approval is only based on studies involving adult patients with HoFH. Further, it frequently causes gastrointestinal symptoms and increases liver fat, at least in the short term, although medium-term follow-up data are hopeful.^46^ Finally, apheresis may not lower LDL-C levels sufficiently in patients with HoFH, as was evident in 7 of the 14 patients in the current trial whose LDL-C remained >130 mg/dL despite regular apheresis before study enrollment, which also continued throughout the study.

During the study period, TEAEs occurred in 71.4% of the patients treated with evinacumab. Overall, evinacumab was generally well-tolerated in these young pediatric patients with HoFH, and its safety profile was consistent with that observed in adult and adolescent patients. Immunogenicity potential was minimal: only one patient developed treatment-emergent ADAs, with a low titer, and no neutralizing antibodies were detected.

This is the first study to assess the effect of a novel monoclonal antibody inhibitor of ANGPTL3 on LDL-C levels in young pediatric patients with HoFH 5 to 11 years of age. Despite their young age, these patients already had a high risk of nonfatal and fatal cardiovascular events, a risk that will only increase with age and inadequate treatment of LDL-C. The 2023 European Atherosclerosis Society Consensus Panel highlights the need for updated diagnostic criteria, early identification of patients with HoFH, cardiac workup at diagnosis and follow-up, family planning, and incorporating novel therapies.^2^

Limitations of this study include the open-label study design, the relatively short follow-up period, and the small sample size, both of which are related to the recruitment constraints of a narrow age range in a rare and high-risk patient population, and relative lack of ethnic diversity. Moreover, long-term follow-up of evinacumab-treated pediatric patients is required to provide definitive evidence of safety. However, no liver abnormalities in adults and adolescent patients with HoFH have been observed with evinacumab over a longer trial duration (>2 years).^47^ This is particularly relevant given the recent discontinuation of the vupanorsen program.^48^ Vupanorsen, an antisense oligonucleotide that inhibits ANGPTL3 protein synthesis, was in part discontinued due to dose-dependent increases in hepatic fat and marked elevations in alanine aminotransferase and aspartate aminotransferase that were observed in a phase 2b trial of adult patients with dyslipidemia.^48^

### Conclusions

The addition of evinacumab to aggressive baseline LLT reduced LDL-C levels by 48.3% in children 5 to 11 years of age with HoFH, with corresponding considerable reductions in other key lipid and lipoprotein parameters. Evinacumab appears to be an effective therapy for patients with HoFH as young as 5 years old, in whom achieving goal LDL-C levels with standard LLTs is not otherwise possible.

## ARTICLE INFORMATION

### Acknowledgments

The authors would like to thank the patients, their families, and all investigators involved in this study. The authors would also like to thank Alpana Waldron, MS, Alison Templeton, PhD, Jian Zhao, MS, and Yi Zhang, PhD, for their contribution toward this study. Medical writing support under the direction of the authors was provided by Rhutika Dessai, MSc, of Prime, London, United Kingdom, funded by Regeneron Pharmaceuticals, Inc, according to Good Publication Practice guidelines (https://www.acpjournals.org/doi/10.7326/M22-1460). The sponsor was involved in the study design and collection, analysis, and interpretation of data, as well as data checking of information provided in the article. All authors had full access to all the data in the study and take responsibility for the integrity of the data and the accuracy of the data analysis. All authors contributed to the concept and design of the study; acquisition, analysis, and interpretation of the data; drafting of the article; and critical revision of the article for important intellectual content. The authors received no honoraria related to the development of this publication.

### Sources of Funding

This study was sponsored by Regeneron Pharmaceuticals, Inc.

### Disclosures

A.W. reports payment or honoraria for lectures, presentations, speakers’ bureaus, article writing, or educational events from Novartis and Algorithm; participation in a data safety monitoring board or advisory board for Amryt Pharma; leadership or fiduciary role in other board, society, committee, or advocacy group, paid or unpaid, for Novartis; and research support for pharmaceutical trials from Amgen, Regeneron Pharmaceuticals, Inc, Novartis, Silence Therapeutics, Esperion, and Ultragenyx. S.G.-P. reports receiving research support from Amgen. S.A., J.M., S.B., P.B., R.T.G., B.H., and R.P. are employees of and stockholders in Regeneron Pharmaceuticals, Inc. E.A.B. is a steering committee member for the REDUCE-IT (Amarin) and PROMINENT (Kowa) trials, and receives research support from Regeneron Pharmaceuticals, Inc. He has received speaking or consulting honoraria from 89Bio, Amarin, Amgen, Amryt, Dalcor, Esperion, Immunovant, Ionis, Merck, New Amsterdam, and Pfizer. M.-J.C. reports honoraria from AstraZeneca, Merck Sharp & Dohme, Pfizer, Amgen, and Sanofi. C.B.-S. reports honoraria for presentations from the National Association of Continuing Medical Education and the Cardiometabolic Health Congress. S.B. reports funding or consulting fees from Amgen, Altimmune, Axcella, Regeneron Pharmaceuticals, Inc, Ionis Pharmaceuticals, Boehringer Ingelheim, Esperion, Lilly, Madrigal, Merck, and Novartis. J.B. reports funding or consulting fees from Regeneron Pharmaceuticals, Inc. P.M.M. reports receipt of research grants to his institution for the participation in the ODYSSEY OUTCOMES trial, as well as financial fees for serving as a medical monitor for the trial and associated support for travel related to trial meetings from Sanofi; consulting fees from Regeneron Pharmaceuticals, Inc, Amgen, Esperion, Kaneka, and Stage II Innovations; advisor fees from Novartis for serving on their advisory committee; and research grants to his institution from Ionis, FH Foundation, GB Life Sciences, Aegerion, Amgen, Kowa, Novartis, and Regeneron Pharmaceuticals, Inc. The other authors report no conflicts.

### Supplemental Material

Study Investigators

Inclusion and Exclusion Criteria

Part B Study End Points

Figures S1–S7

Tables S1–S3

## Supplementary Material
