# Supplementary material for: Evinacumab for Pediatric Patients With Homozygous Familial Hypercholesterolemia
Source: Circulation. 2023 Oct 20;149(5):343–53. doi: 10.1161/CIRCULATIONAHA.123.065529 (PMC10814999; doi:10.1161/CIRCULATIONAHA.123.065529)
Supplement: Supplementary file 1 [file cir-149-343-s001.pdf]

SUPPLEMENTAL MATERIAL

Study Investigators

Australia: Shubha Srinivasan (Sydney)

Austria: Susanne Greber-Platzer (Vienna)

Taiwan: Min-Ji Charng (Taipei)

The Netherlands: Albert Wiegman (Amsterdam)

USA: Carissa Baker-Smith (Delaware), Seth Baum (Florida), Eliot A. Brinton (Utah), Julie Brothers (Pennsylvania), Jacob Hartz (Massachusetts), Patrick Moriarty (Kansas)

Inclusion and Exclusion Criteria

Inclusion Criteria

Patients meeting the below-mentioned criteria were included in the study:

- Males and females aged 5–11 years at the time of the screening visit.
- Diagnosis of functional homozygous familial hypercholesterolemia by either genetic or clinical criteria.
  - Genetic criteria included:
    - Documented functional pathogenic variants in both low-density lipoprotein receptor (*LDLR*) alleles. Patients who had negative receptor mutations on both LDLR alleles (i.e., were double-negative) were eligible.
    - Documented homozygous pathogenic variants in LDLR adaptor protein 1, or homozygous or compound heterozygous pathogenic variant in the apolipoprotein B (*APOB*) or proprotein convertase subtilisin/kexin type 9 (*PCSK9*) genes. Patients who were double heterozygous, i.e., those with pathogenic variants on different genes (*LDLR/PCSK9* or *LDLR/APOB*), were also eligible.
  - Clinical criteria included:
    - Untreated total cholesterol (TC) >500 mg/dL (>13 mmol/L), triglycerides (TGs) <300 mg/dL (<7.8 mmol/L), and both parents with documented TC >250 mg/dL, or presence of cutaneous or tendinous xanthoma in the study patient before the age of 10 years.

- LDL-C levels >130 mg/dL at the screening visit.
- Body weight ≥15 kg.
- Patients receiving stable maximally tolerated therapy (maximally tolerated therapy could include a daily statin) at the screening visit were included. Those who were not able to be on a maximum daily statin had to be on an appropriate dose or no statin, according to the investigator's judgment.
- Patients had to be willing and able to comply with clinic visits and study-related procedures.
- Parent(s) or legal guardian(s) had to provide the signed informed consent form (ICF). Patients aged ≥5 years had to also provide informed assent forms (IAF) to enroll in the study, and provide a separate IAF or ICF signed and dated by the parent(s)/legal guardian(s) (as appropriate based on local regulations and requirements).

Exclusion Criteria

A patient who met any of the following criteria was excluded from the study:

- Background pharmacologic medical lipid-modifying therapy, nutraceuticals, or over-the-counter therapy known to affect lipids, at a dose/regimen that had not been stable for at least 4 weeks (8 weeks for PCSK9 inhibitors) before the screening visit.
- Unwilling to enter the run-in period.
- For patients entering Part A, unable to temporarily discontinue apheresis from the baseline visit through the week 4 visit.

- For those receiving lipid apheresis, having a setting (if applicable) and schedule that had not been stable for approximately 8 weeks before the screening visit, or having an apheresis schedule that is not anticipated to be stable over the duration of the treatment period (48 weeks). A stable schedule was defined as weekly (every 7±1 days) or every other week (every 14±2 days).
- Plasmapheresis within 8 weeks of the screening visit, or plans to undergo plasmapheresis during Part A or Part B.
- Presence of any clinically significant uncontrolled endocrine disease known to influence serum lipids or lipoproteins.
- Newly diagnosed (within 3 months prior to randomization visit [week 0/day 1]) diabetes mellitus or poorly controlled (hemoglobin A1c >9%) diabetes.
- Chronic use of systemic corticosteroids, unless used as replacement therapy for pituitary/adrenal disease with a stable regimen for at least 6 weeks prior to randomization (topical, intra-articular, nasal, inhaled, and ophthalmic steroid therapies were not considered as 'systemic' and were allowed).
- History of a myocardial infarction, percutaneous coronary intervention, uncontrolled cardiac arrhythmia, carotid surgery or stenting, stroke, transient ischemic attack, valve replacement surgery, carotid revascularization, endovascular procedure, or surgical intervention for peripheral vascular disease within 3 months prior to the screening visit.
- History of cancer within the past 5 years.
- Use of any active investigational drugs within 1 month or 5 half-lives, whichever is

longer.

- Conditions/situations such as:

  – Any clinically significant abnormality identified at the time of screening that, in the judgment of the investigator or any sub-investigator, would preclude safe completion of the study or constrain endpoints assessment, eg, major systemic diseases or patients with short life expectancy.

  – Considered by the investigator or any sub-investigator as inappropriate for this study for any reason, eg, deemed unable to meet specific protocol requirements such as scheduled visits; or an investigator or any sub-investigator, pharmacist, study coordinator, other study staff, or relative thereof directly involved in the conduct of the protocol, etc.

  – Presence of any other conditions (eg, geographic or social), either actual or anticipated, that the investigator feels would restrict or limit the patient's participation for the duration of the study.

- Laboratory findings during the screening period (not including randomization labs): Positive urine pregnancy test in females of childbearing potential, TGs >300 mg/dL (>4.52 mmol/L; 1 repeat lab is allowed), alanine aminotransferase or aspartate aminotransferase >3 times the upper limit of normal (ULN; 1 repeat lab is allowed), creatine phosphokinase >3 times the ULN (1 repeat lab is allowed).

- Known hypersensitivity to monoclonal antibodies or any excipient in the evinacumab solution for infusion.

- Sexually active males and sexually active females of childbearing potential at screening.

- Female patients who have commenced menstruating at any time during the study and are found to have a positive urine pregnancy test, or who are sexually active but not using or not willing to use an established highly effective contraception method prior to subsequent dosing of study treatment, during the study, and for at least 24 weeks after the last dose of the study drug. Highly effective contraceptive measures include:
  - Stable use of combined (estrogen- and progestogen-containing) hormonal contraception associated with inhibition of ovulation initiated 2 or more menstrual cycles prior to screening: oral, intravaginal, or transdermal.
  - Stable use of progestogen-only hormonal contraception associated with inhibition of ovulation initiated 2 or more menstrual cycles prior to screening: oral, injectable, or implantable. Including the use of the intrauterine device or the intrauterine hormone-releasing system. Use of bilateral tubal ligation, or having a vasectomized male partner. Vasectomized partner is a highly effective birth control method provided that the partner is the sole male sexual partner of the female of the childbearing potential trial participant, and that the vasectomized partner has received a medical assessment of the surgical success.

  - Sexual abstinence is considered a highly effective method only if defined as refraining from heterosexual intercourse during the entire period of risk associated with study treatments. True abstinence: When this was in line with the preferred and usual lifestyle of the patient. Periodic abstinence (calendar, symptothermal, and post-ovulation methods), withdrawal (coitus interruptus), spermicides only, and the lactational amenorrhea method are not acceptable methods of contraception.

- Male patients who are sexually active at any time during the study and unwilling to use established acceptable contraception methods of consistent use of barrier contraception with spermicide during the study and for up to 24 weeks after the last infusion of the study drug.

- Individuals who were accommodated in an institution by official or court order.

Part B Study Endpoints

Primary Endpoint

- The percent change in calculated LDL-C from baseline to week 24 (ITT estimand) in Part B. The primary endpoint was defined as: 100 x (calculated LDL-C value at week 24 – calculated LDL-C value at baseline)/calculated LDL-C value at baseline.

- The baseline LDL-C value was defined as the last calculated LDL-C value obtained before the first dose of study treatment in Part B. The calculated LDL-C measurement at week 24 will be the LDL-C value obtained within the week 24 analysis window, regardless of adherence to treatment and subsequent therapies (ITT estimand).

- All calculated LDL-C values (scheduled or unscheduled, fasting or not fasting) may be used to provide a value for the primary efficacy endpoint, if appropriate, according to the above definition.

Secondary Endpoints

- The percent change in ApoB from baseline to week 24 (ITT estimand).

- The percent change in non-high-density lipoprotein cholesterol (non-HDL-C) from baseline to week 24 (ITT estimand).

- The percent change in TC from baseline to week 24 (ITT estimand).

- The proportion of patients with ≥50% reduction in calculated LDL-C at week 24 (ITT estimand).

- The percent change in calculated LDL-C from baseline to week 24 in patients who have defective and negative pathogenic variants (ITT estimand).
- The percent change in lipoprotein (a) from baseline to week 24 (ITT estimand).
- The absolute change in LDL-C at week 24 (ITT estimand).
- Incidence of treatment-emergent adverse events and other safety variables over time.
- Concentrations of total evinacumab over time.
- Pharmacokinetic parameters including steady-state maximum concentration, steady-state area under the concentration-time profile of dosing interval, and steady state trough concentration.
- Incidence and titer of treatment-emergent anti-drug antibodies over time.

Figure S1. Study Design of Parts A to C.

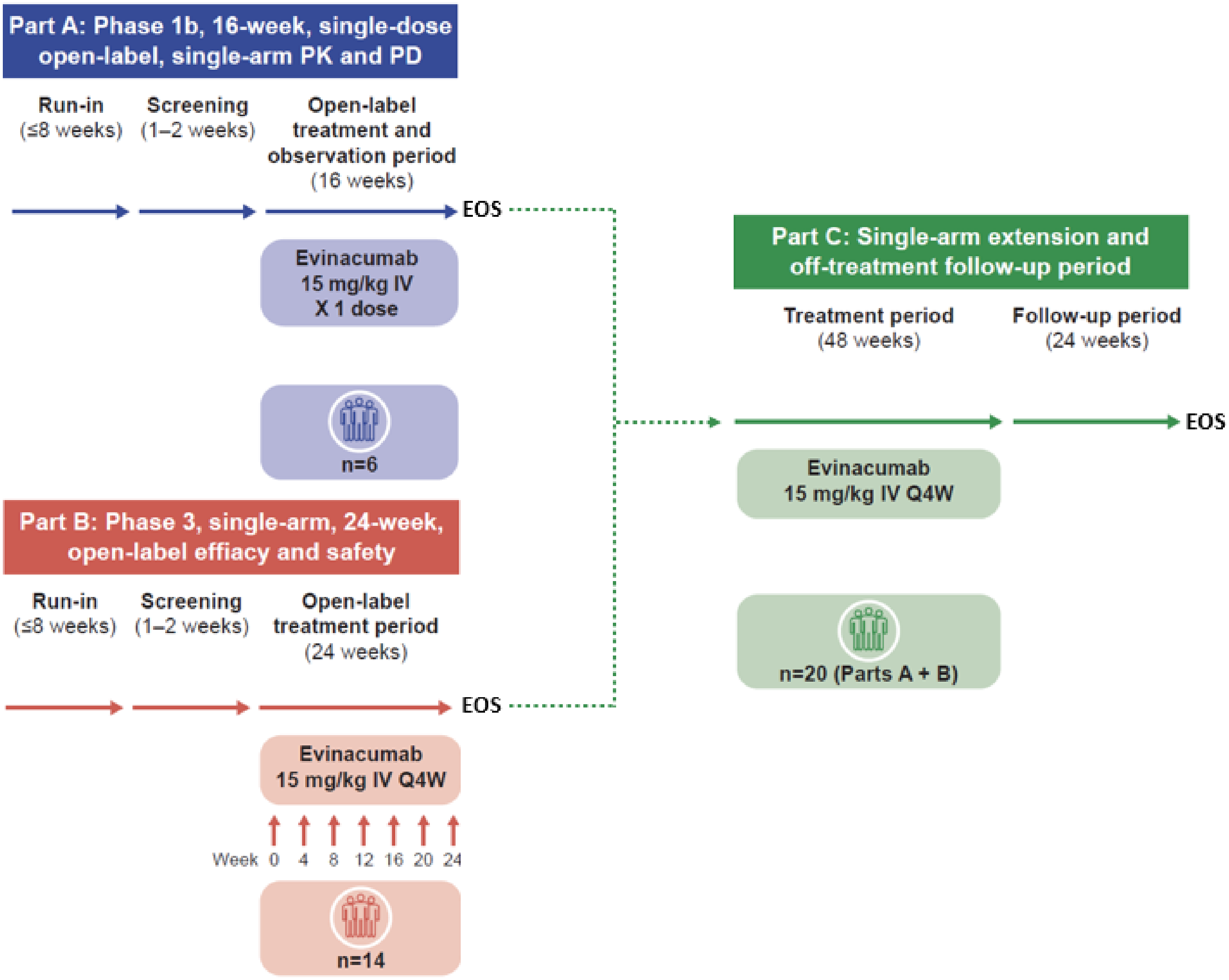

EOS indicates end of study; IV, intravenous; PD, pharmacodynamics; PK, pharmacokinetics; and Q4W, every 4 weeks.

Figure S2. Mean Change in LDL-C From Baseline Over Time After a Single Dose of Evinacumab in Part A (Safety Set Analysis).

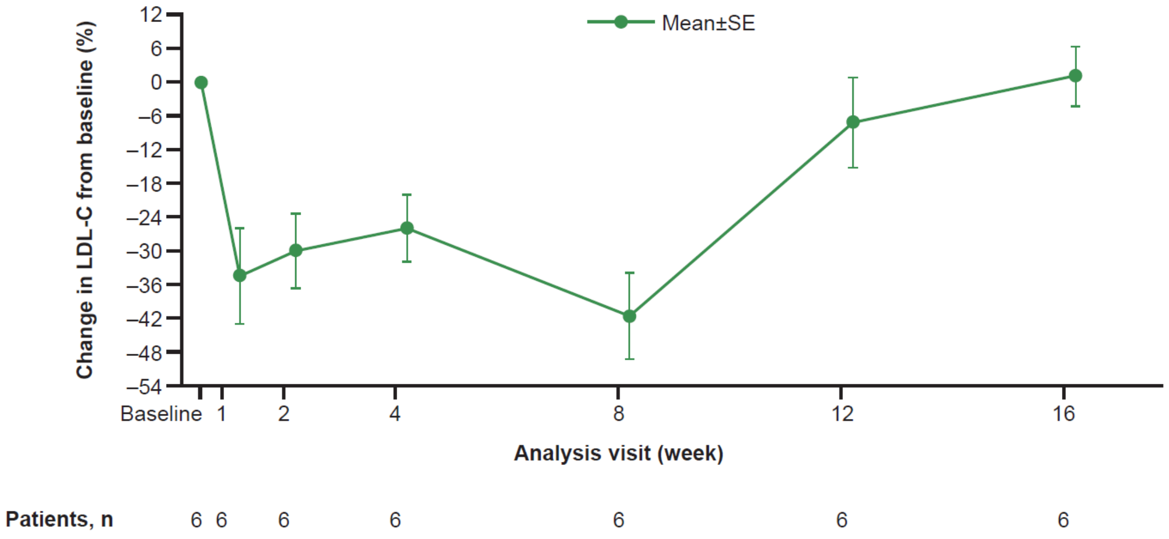

LDL-C indicates low-density lipoprotein cholesterol, and SE, standard error.

Figure S3. Patient Disposition From Part B of the Study.

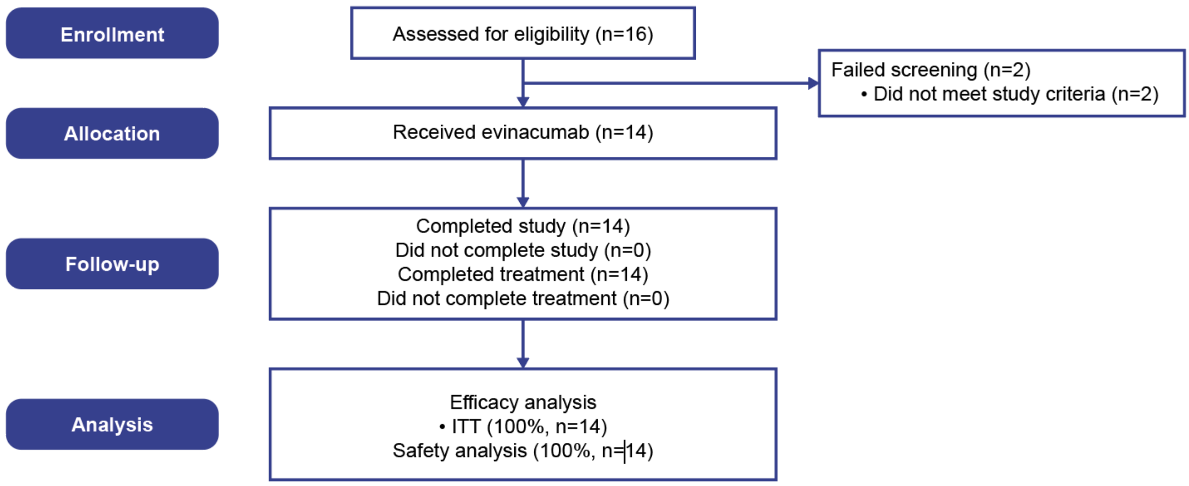

All 14 enrolled patients received evinacumab every 4 weeks until week 24 (until the end of the study).

ITT indicates intent-to-treat.

Figure S4. (A) Total Evinacumab Concentration Over Time and (B) Mean Percent Change in LDL-C from Baseline at Week 24 in Individual Patients from Part B.

A.

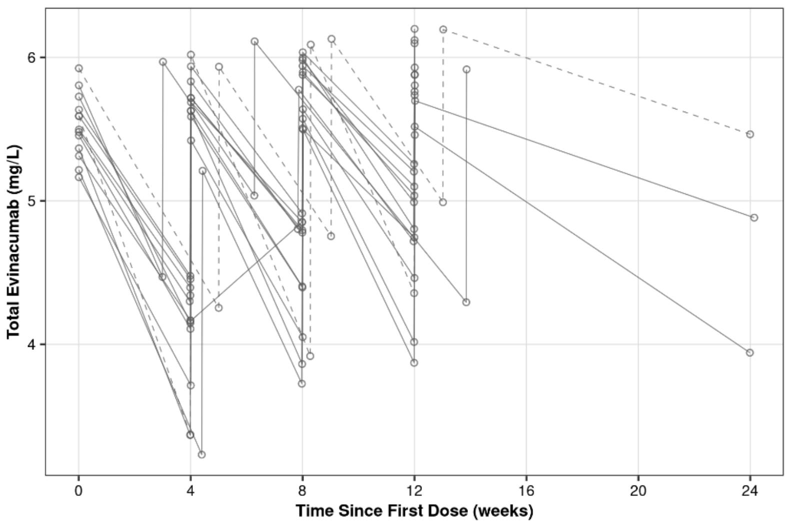

B.

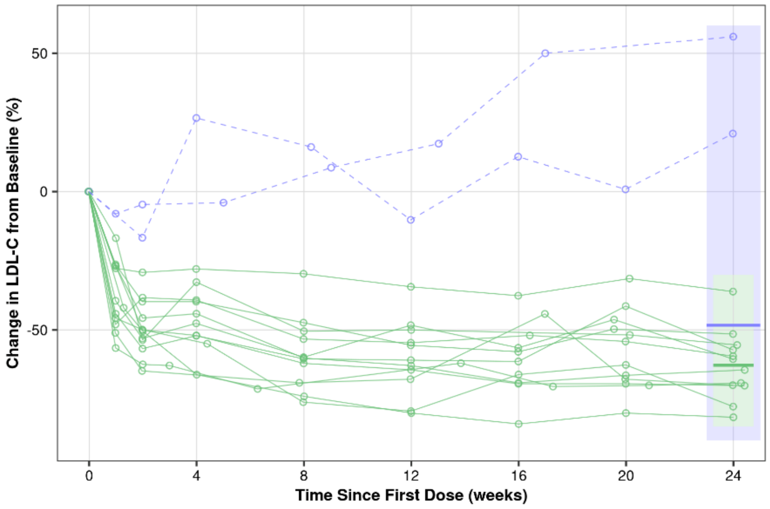

A. The dashed lines represent individuals in which treatment with evinacumab did not result in reductions in LDL-C.

B. Individual data are shown in blue (dashed lines) for 2 participants for whom LDL-C generally was generally not reduced from baseline and in green (solid lines) for the remaining participants. The thick blue and green horizontal lines around 24 weeks represent the mean percent change in LDL-C from baseline at week 24 for all participants (blue shaded area) or excluding participants in which LDL-C was not reduced from baseline (green shaded area).

LDL-C indicates low-density lipoprotein cholesterol.

Figure S5. Mean Percent Change in LDL-C From Baseline at Week 24 in Individual Patients from Part B of the Study (ITT Population).

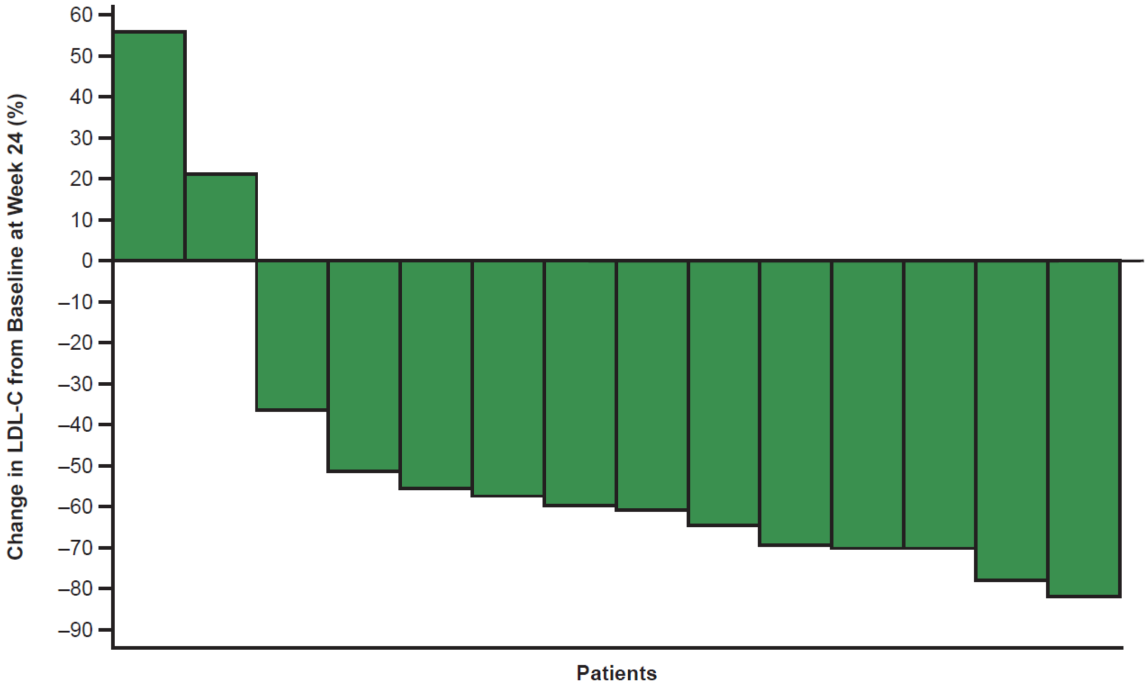

ITT indicates intent-to-treat; and LDL-C, low-density lipoprotein cholesterol.

Figure S6. Percent Change from Baseline in LDL-C at Week 24 in Part B According to Baseline Subgroups (ITT Population).

	n	Combined mean (95% CI)
**Overall**	14	−48.3 (−68.8 to −27.8)
**Sex**		
Male	6	−31.4 (−75.6 to 12.9)
Female	8	−61 (−71.3 to −50.8)
**Age (years)**		
≥5 to <10	7	−65.7 (−72.3 to −59.2)
≥10 to <12	7	−30.9 (−68.1 to 6.3)
**Race**		
White	8	−67 (−73.6 to −60.4)
Black or African American	1	56 (N/A)
Asian	2	−56 (−64.9 to −47.1)
American Indian or Alaska Native	1	21 (N/A)
Other	2	−52.7 (−85.1 to −20.3)
**Ethnicity**		
Not Hispanic or Latino	13	−46.7 (−68.6 to −24.9)
Not reported	1	−69.2 (N/A)
**Baseline apheresis status**		
Yes	7	−47.9 (−72.3 to −23.4)
No	7	−48.8 (−83.7 to −13.9)
**Receptor-negative mutation in both *LDLR* activity**		
Negative/negative	4	−45.5 (−89.9 to −1.2)
Negative/defective	5	−66.6 (−75.1 to −58.0)
Defective/defective	5	−32.3 (−76.8 to 12.3)

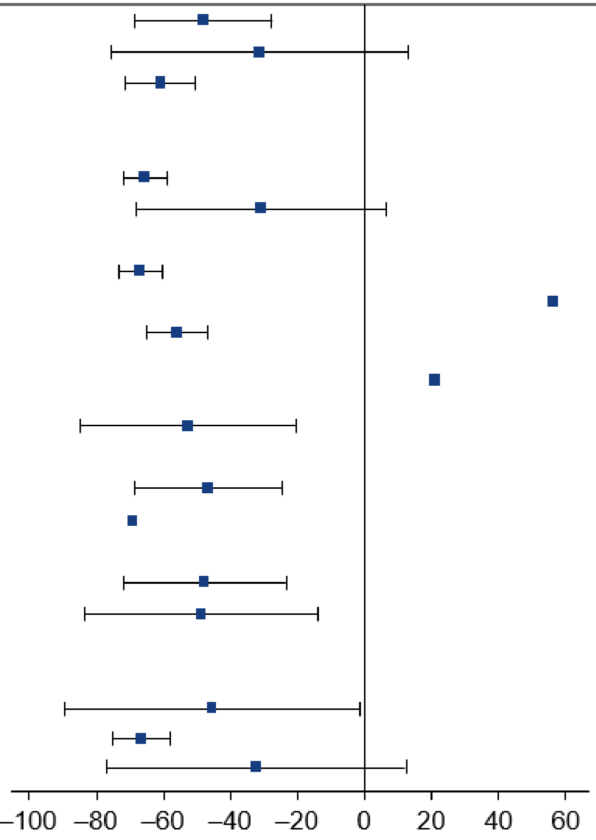

CI indicates confidence interval; ITT, intent-to-treat; LDL-C, low-density lipoprotein cholesterol; and LDLR, low-density lipoprotein receptor.

Figure S7. Percent Change in (A) ApoB, (B) non-HDL-C, (C) Total Cholesterol, and (D) Lp(a) at Week 24 (ITT Population).

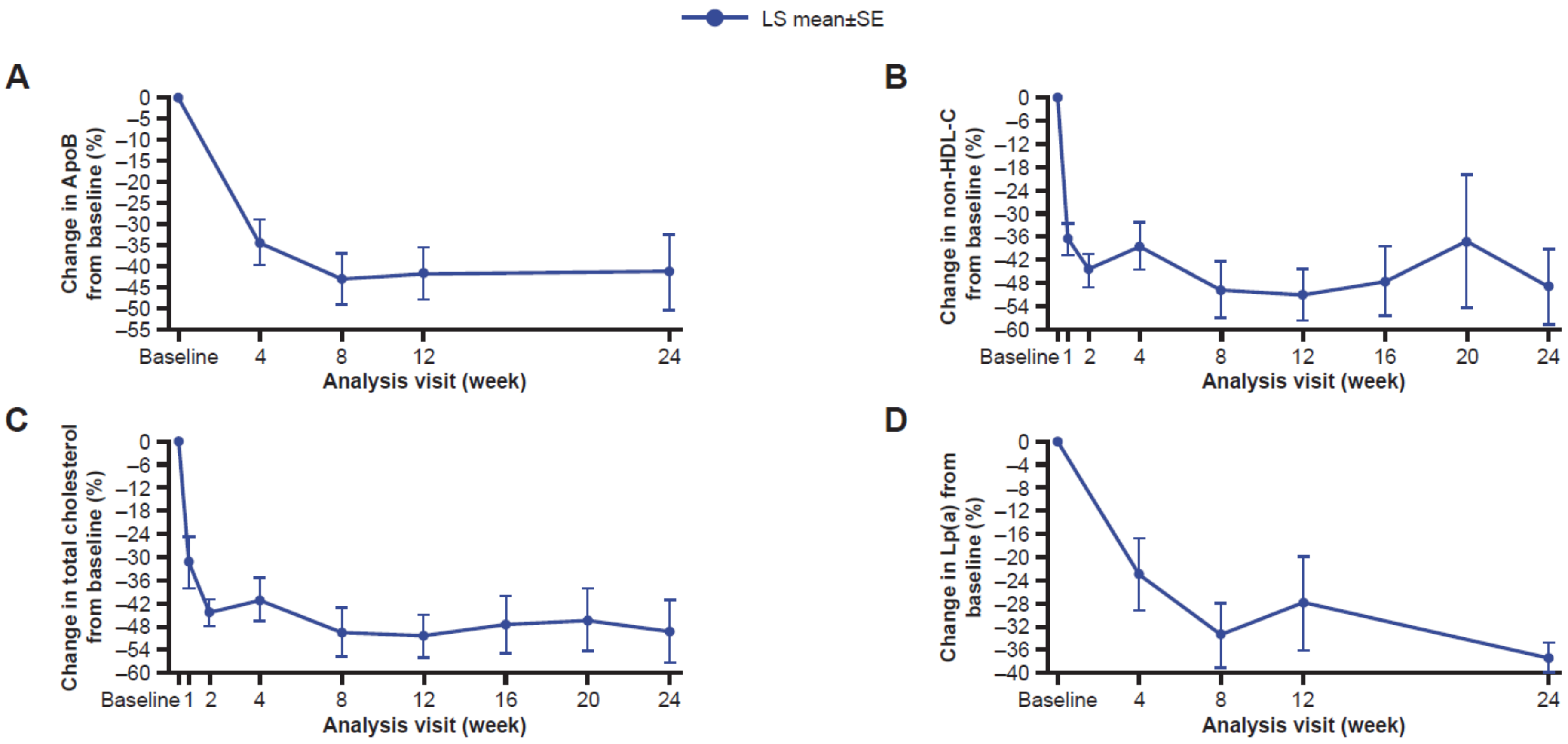

ApoB indicates apolipoprotein B; ITT, intent-to-treat; LDL-C, low-density lipoprotein cholesterol; Lp(a), lipoprotein (a); non-HDL-C, non-high-density lipoprotein cholesterol; and SE, standard error.

Table S1. *LDLR* Genotype and Variant Categorization for Part B (ITT Population).

Parameter, n (%)	Part B (n=14)
Genotype state	
Homozygous (*LDLR*)	4 (28.6)
Compound heterozygous (*LDLR*)	10 (71.4)
LDLR variant category	
Defective/defective	5 (35.7)
Defective/negative	5 (35.7)
Negative/negative	4 (28.5)

ITT indicates intent-to-treat; and LDLR, low-density lipoprotein receptor.

Table S2. Change from Baseline in LDL-C at Week 24 in Part B According to Baseline LDL-C Median Values and Lomitapide Treatment (ITT Population).

Parameter	Part B (n=14)
Baseline LDL-C median values, mg/dL	
≥Median, n	7
Mean±SE (95% CI)	−54.0±13.7 (−80.9 to −27.2)
<Median, n	7
Mean±SE) (95% CI)	−42.6±16.6 (−75.1 to −10.1)
Lomitapide treatment, %	
Yes, n	2
Mean±SE (95% CI)	−28.4±49.3) (−125.1 to 68.3)
No, n	12
Mean±SE (95% CI)	−51.6±10.3 (−71.9 to −31.4)

CI indicates confidence interval, ITT intent-to-treat, LDL-C, low-density lipoprotein cholesterol, and SE, standard error.

Table S3. Change from Baseline in Lp(a) at Week 24 in Part B According to Baseline Apheresis Status (ITT Population).

Parameter	Part B (n=14)
Baseline apheresis status	
Yes, n	7
Mean±SE (95% CI), %	−43.7±1.6 (−46.8 to −40.6)
No, n	7
Mean±SE (95% CI), %	−32.7±1.4 (−35.4 to −30.1)

CI indicates confidence interval, ITT intent-to-treat, Lp(a), lipoprotein (a), and SE, standard error.
