# Supplementary material for: Evinacumab for Pediatric Patients With Homozygous Familial Hypercholesterolemia
Source: Circulation. 2023 Oct 20;149(5):343–53. doi: 10.1161/CIRCULATIONAHA.123.065529 (PMC10814999; doi:10.1161/CIRCULATIONAHA.123.065529)
Supplement: Supplementary file 2 [file cir-149-343-s002.pdf]

SUPPLEMENTAL MATERIAL

	Pre-*CPR-NOVA* (N = 96)	*CPR-NOVA* (N = 138)	p
Demographics			
Age			0.128
< 1 months	26 (27.1%)	20 (14.5%)	
1 months - < 1 year	37 (38.5%)	59 (42.8%)	
1 year - < 12 years	23 (24.0%)	40 (29.0%)	
> 12 years	10 (10.4%)	19 (13.8%)	
Weight (kg)	6.1 [3.6, 15.0]	7.5 [4.4, 20.0]	0.144
Male	54 (56.3%)	63 (45.7%)	0.144
Race			0.018
White	55 (57.3%)	63 (45.7%)	
Black or African American	16 (16.7%)	46 (33.3%)	
Other	5 (5.2%)	5 (3.6%)	
Unknown or Not Reported	20 (20.8%)	24 (17.4%)	
Hispanic or Latino	14 (14.6%)	21 (15.2%)	1.000
Pre-existing medical conditions			
Respiratory insufficiency	81 (84.4%)	124 (89.9%)	0.231
Hypotension	79 (82.3%)	88 (63.8%)	0.002
Heart failure	13 (13.5%)	21 (15.2%)	0.851
Pneumonia	9 (9.4%)	14 (10.1%)	1.000
Sepsis	15 (15.6%)	22 (15.9%)	1.000
Trauma	0 (0.0%)	5 (3.6%)	0.080
Kidney injury / disease	11 (11.5%)	27 (19.6%)	0.108
Malignancy	2 (2.1%)	7 (5.1%)	0.315
Pulmonary hypertension	20 (20.8%)	24 (17.4%)	0.610
Congenital heart disease	68 (70.8%)	83 (60.1%)	0.098
Pre-event characteristics			
Illness category			0.028
Medical cardiac	30 (31.3%)	35 (25.4%)	
Medical non-cardiac	20 (20.8%)	44 (31.9%)	
Surgical cardiac	44 (45.8%)	46 (33.3%)	
Surgical non-cardiac	2 (2.1%)	8 (5.8%)	
Trauma	0 (0.0%)	5 (3.6%)	
PRISM*	8.0 [3.0, 13.5]	5.0 [0.0, 11.0]	0.018
Vasoactive-inotrope score†	3.0 [0.0, 10.0]	0.0 [0.0, 8.0]	0.040
Baseline PCPC score‡			0.624
1 - Normal	59 (61.5%)	82 (59.4%)	
2 - Mild disability	16 (16.7%)	22 (15.9%)	

3 - Moderate disability	12 (12.5%)	16 (11.6%)	
4 - Severe disability	7 (7.3%)	17 (12.3%)	
5 - Coma/vegetative state	2 (2.1%)	1 (0.7%)	
Baseline FSS‡	6 [6, 9]	6 [6, 10]	0.193

Table S1: Patient Characteristics by Enrollment Period. Comparison of characteristics between patients enrolled in the *ICU-RESUS* parent study prior to prospective collection of ETCO2 waveform data for C*PR-NOVA* study (pre-*CPR-NOVA*: October 1, 2016 through May 5, 2019) versus those enrolled after the start of the *CPR-NOVA* ancillary investigation (May 6, 2019 through March 31, 2021).

Analyses performed with Fisher's exact test, Wilcoxon rank-sum test, or Cochrane-Armitage trend test.

$ETCO_2$ indicates end-tidal carbon dioxide; FSS, Functional Status Scale; PCPC, Pediatric Cerebral Performance Category; PRISM, Pediatric RISk of Mortality score.

* PRISM was evaluated 2 - 6 hours prior to the event.

† Vasoactive inotrope score was evaluated 2 hours prior to the event and calculated from the following equation: dopamine dose (μg/kg/min) + dobutamine dose (μg/kg/min) + nitroprusside dose (μg/kg/min) + (10 × milrinone dose (μg/kg/min)) + (100 × epinephrine dose (μg/kg/min)) + (100 × norepinephrine dose (μg/kg/min)) + (100 × phenylephrine dose (μg/kg/min)) + vasopressin dose (munits/kg/hr).

‡ Baseline PCPC and FSS represent patient status prior to the event leading to hospitalization.

	Survival to Hospital Discharge		
	Yes (N = 134)	No (N = 100)	p
Demographics			
Age			0.020
≤ 1 year	90 (67.2%)	52 (52.0%)	
1 year - < 8 years	27 (20.1%)	22 (22.0%)	
8 years - < 19 years	17 (12.7%)	26 (26.0%)	
Weight (kg)	6.2 [3.8,11.4]	8.3 [4.6,23.4]	0.039
Male	68 (50.7%)	49 (49.0%)	0.895
Race			0.854
White	65 (48.5%)	53 (53.0%)	
Black or African American	32 (23.9%)	30 (30.0%)	
Other	5 (3.7%)	5 (5.0%)	
Unknown or Not Reported	32 (23.9%)	12 (12.0%)	
Hispanic or Latino	22 (16.4%)	13 (13.0%)	0.460
Pre-existing conditions			
Respiratory insufficiency	124 (92.5%)	81 (81.0%)	0.009
Hypotension	85 (63.4%)	82 (82.0%)	0.002
Heart failure	11 (8.2%)	23 (23.0%)	0.002
Pneumonia	10 (7.5%)	13 (13.0%)	0.186
Sepsis	10 (7.5%)	27 (27.0%)	<0.001
Trauma	3 (2.2%)	2 (2.0%)	1.000
Kidney injury / disease	11 (8.2%)	27 (27.0%)	<.001
Malignancy	2 (1.5%)	7 (7.0%)	0.040
Pulmonary hypertension	22 (16.4%)	22 (22.0%)	0.312
Congenital heart disease	87 (64.9%)	64 (64.0%)	0.891
Pre-event characteristics			
Illness category			0.653
Medical cardiac	35 (26.1%)	30 (30.0%)	
Medical non-cardiac	34 (25.4%)	30 (30.0%)	
Surgical cardiac	57 (42.5%)	33 (33.0%)	
Surgical non-cardiac	5 (3.7%)	5 (5.0%)	
Trauma	3 (2.2%)	2 (2.0%)	
PRISM[1]	5.0 [0.0, 10.0]	8.5 [3.0,1 4.0]	0.004
Vasoactive-inotrope score[†]	0.0 [0.0, 6.9]	3.0 [0.0, 10.8]	0.006
Baseline PCPC score[‡]			0.030
1 - Normal	89 (66.4%)	52 (52.0%)	
2 - Mild disability	20 (14.9%)	18 (18.0%)	
3 - Moderate disability	12 (9.0%)	16 (16.0%)	

	Survival to Hospital Discharge		
	Yes (N = 134)	No (N = 100)	p
4 - Severe disability	12 (9.0%)	12 (12.0%)	
5 - Coma/vegetative state	1 (0.7%)	2 (2.0%)	
Baseline FSS‡	6.0 [6.0, 8.0]	7.0 [6.0, 11.0]	0.014

Table S2: Patient Characteristics by Survival. Comparison of characteristics between patients with survival to hospital discharge versus those that did not survive. Analyses performed with Fisher's exact test, Wilcoxon rank-sum test, or Cochrane-Armitage trend test.

$ETCO_2$ indicates end-tidal carbon dioxide; FSS, Functional Status Scale; PCPC, Pediatric Cerebral Performance Category; PRISM, Pediatric RISk of Mortality score.

* PRISM was evaluated 2 - 6 hours prior to the event.

† Vasoactive-inotrope score was evaluated 2 hours prior to the event and calculated from the following equation: dopamine dose (μg/kg/min) + dobutamine dose (μg/kg/min) + nitroprusside dose (μg/kg/min) + (10 × milrinone dose (μg/kg/min)) + (100 × epinephrine dose (μg/kg/min)) + (100 × norepinephrine dose (μg/kg/min)) + (100 × phenylephrine dose (μg/kg/min)) + vasopressin dose (munits/kg/hr).

‡ Baseline PCPC and FSS represent patient status prior to the event leading to hospitalization.

	Survival to Hospital Discharge		
	Yes (N = 134)	No (N = 100)	p
Interventions in place prior to event			
Central venous catheter	98 (73.1%)	81 (81.0%)	0.212
Vasoactive infusion	72 (53.7%)	79 (79.0%)	<0.001
Invasive mechanical ventilation	119 (88.8%)	93 (93.0%)	0.366
Non-invasive ventilation	9 (6.7%)	7 (7.0%)	1.000
Immediate cause(s) of event			
Arrhythmia	19 (14.2%)	13 (13.0%)	0.849
Cyanosis without respiratory decompensation	4 (3.0%)	5 (5.0%)	0.502
Hypotension	75 (56.0%)	71 (71.0%)	0.021
Respiratory decompensation	62 (46.3%)	36 (36.0%)	0.141
Duration of CPR (minutes)	5.0 [2.0,12.0]	21.5 [7.0,43.0]	<0.001
CPR timing*			0.717
Weekday	76 (56.7%)	61 (61.0%)	
Weeknight	27 (20.1%)	16 (16.0%)	
Weekend	31 (23.1%)	23 (23.0%)	
Medications during CPR			
Epinephrine	104 (77.6%)	94 (94.0%)	<0.001
Number of doses	2 [1, 3]	4 [2, 8]	<0.001
Average interval between doses†	5.3 [4.0, 11.0]	4.4 [3.4, 7.3]	0.035
Time to first dose	1.0 [0.0, 2 .0]	1.0 [0.0, 2.0]	0.713
Atropine	9 (6.7%)	7 (7.0%)	1.000
Calcium	45 (33.6%)	63 (63.0%)	<0.001
Sodium bicarbonate	53 (39.6%)	69 (69.0%)	<0.001
Vasopressin	3 (2.2%)	7 (7.0%)	0.103
Amiodarone	5 (3.7%)	3 (3.0%)	1.000
Lidocaine	4 (3.0%)	2 (2.0%)	1.000
Fluid bolus	22 (16.4%)	36 (36.0%)	<0.001

Table S3: Cardiac Arrest Event Characteristics by Survival. Comparison of cardiac arrest event characteristics between patients between patients with survival to hospital discharge versus those that did not survive. Analyses performed with Fisher's exact test or Wilcoxon rank-sum test. ETCO2 indicates end-tidal carbon dioxide; CPR, cardiopulmonary resuscitation.

* Weekday is between 7 AM and 11 PM Monday - Friday; weeknight, after 11 PM Monday - Thursday; weekend, 11 PM on Friday through 7 AM on the following Monday.

† (total duration of CPR in minutes – time to first epinephrine dose in minutes) / (total number of epinephrine doses – 1)

	ETCO2 ≥20 mmHg	ETCO2 <20 mmHg	p
Highest lactate (mmol/L; first 6 hours post-ROC)*	5.7 [2.4, 11.6]	7.5 [4.7, 14.8]	0.010
Lowest pH (first 6 hours post-ROC)*	7.25 [7.16, 7.34]	7.25 [7.12, 7.32]	0.309
EEG Background Category			0.139
Normal	6/35 (17.1%)	9/29 (31.0%)	
Slow/Disorganized	19/35 (54.3%)	8/29 (27.6%)	
Discontinuous/Burst Suppression	9/35 (25.7%)	9/29 (31.0%)	
Attenuated	1/35 (2.9%)	3/29 (10.3%)	
Vasoactive-inotrope score (at 6 hours post-ROC)†	2.5 [0.0, 9.5]	3.0 [0.0, 10.0]	0.680

Table S4: Post-arrest outcomes. Comparison of tertiary post-arrest outcomes between patients with average event-level ETCO2 ≥20 mmHg versus those with lower ETCO2 values. Analyses performed with Fisher's exact test or Wilcoxon rank-sum test.

$ETCO_2$ indicates end-tidal carbon dioxide; ROC, return of circulation (return of spontaneous circulation or ROC via extracorporeal membrane oxygenation CPR; EEG, electroencephalogram
* Highest arterial or venous lactate and lowest arterial or venous pH in first 6 hours after return of circulation from either venous or arterial sample.
† Calculated from the following equation: dopamine dose (μg/kg/min) + dobutamine dose (μg/kg/min) + nitroprusside dose (μg/kg/min) + (10 × milrinone dose (μg/kg/min)) + (100 × epinephrine dose (μg/kg/min)) + (100 × norepinephrine dose (μg/kg/min)) + (100 × phenylephrine dose (μg/kg/min)) + vasopressin dose (munits/kg/hr).

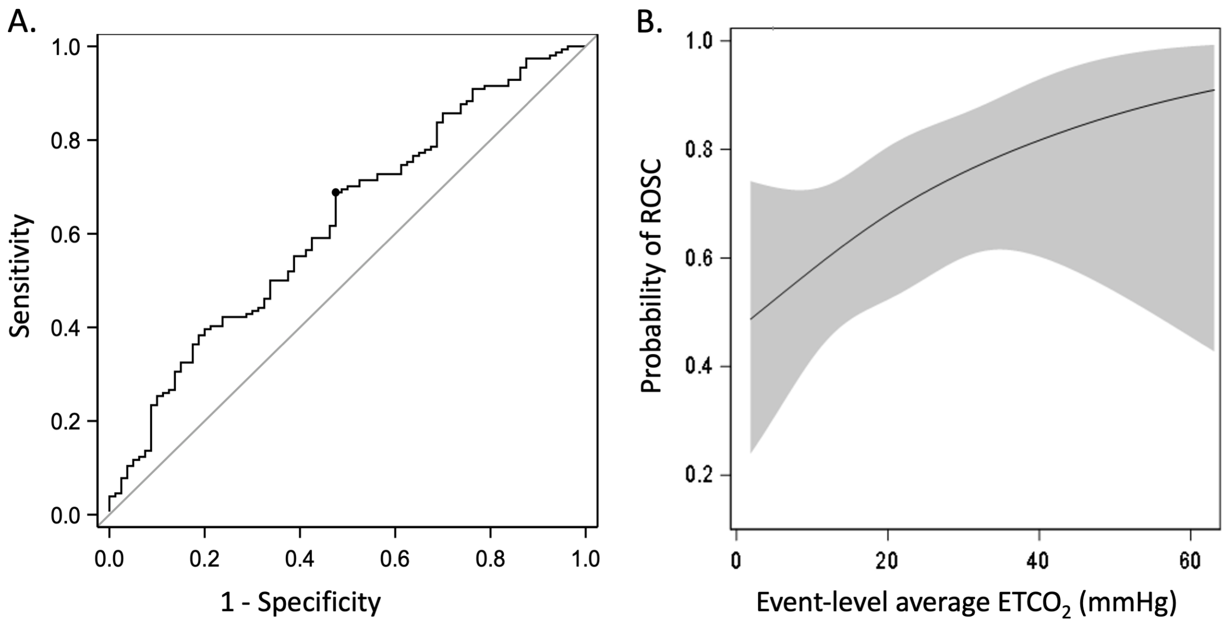

Figure S1: Relationship Between ETCO2 and Return of Spontaneous Circulation (Panel A: Receiver Operating Characteristic (ROC) Curve; Panel B: Probability Curves). ROC curve: optimal cut point: 19.07 mmHg; sensitivity 0.69, specificity 0.53; AUC 0.62 [CI95 0.55 – 0.7]). Probability curves were created with logistic regression and natural cubic splines, controlling for initial rhythm (pulseless vs. non-pulseless), and from respiratory decompensation and arrhythmia as the immediate causes of arrest.

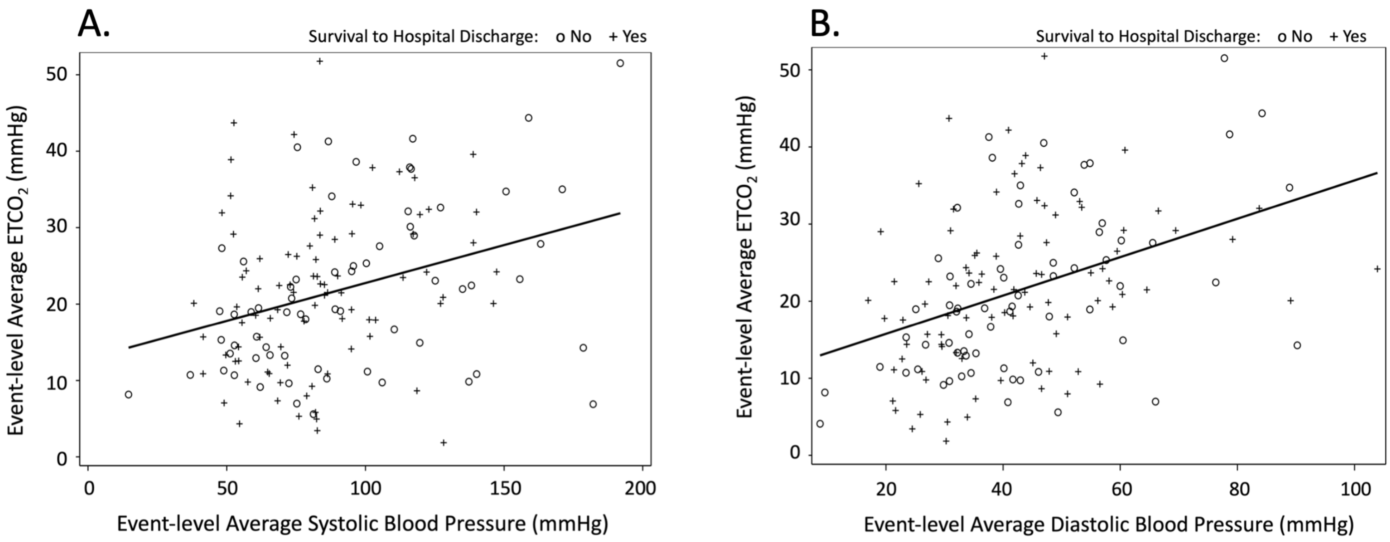

Figure S2: Scatterplot of ETCO2 vs. Systolic (Panel A) and Diastolic (Panel B) Blood Pressure (BP). Each marker represents the event-level average ETCO2 and BP. The line represents the least squares regression line (systolic BP regression equation: event-level average ETCO2 = 12.8 + 0.1 * event-level average systolic BP, $p<0.01$; diastolic BP regression equation: event-level average ETCO2 = 10.8 + 0.2 * event-level average diastolic BP, $p<0.01$).

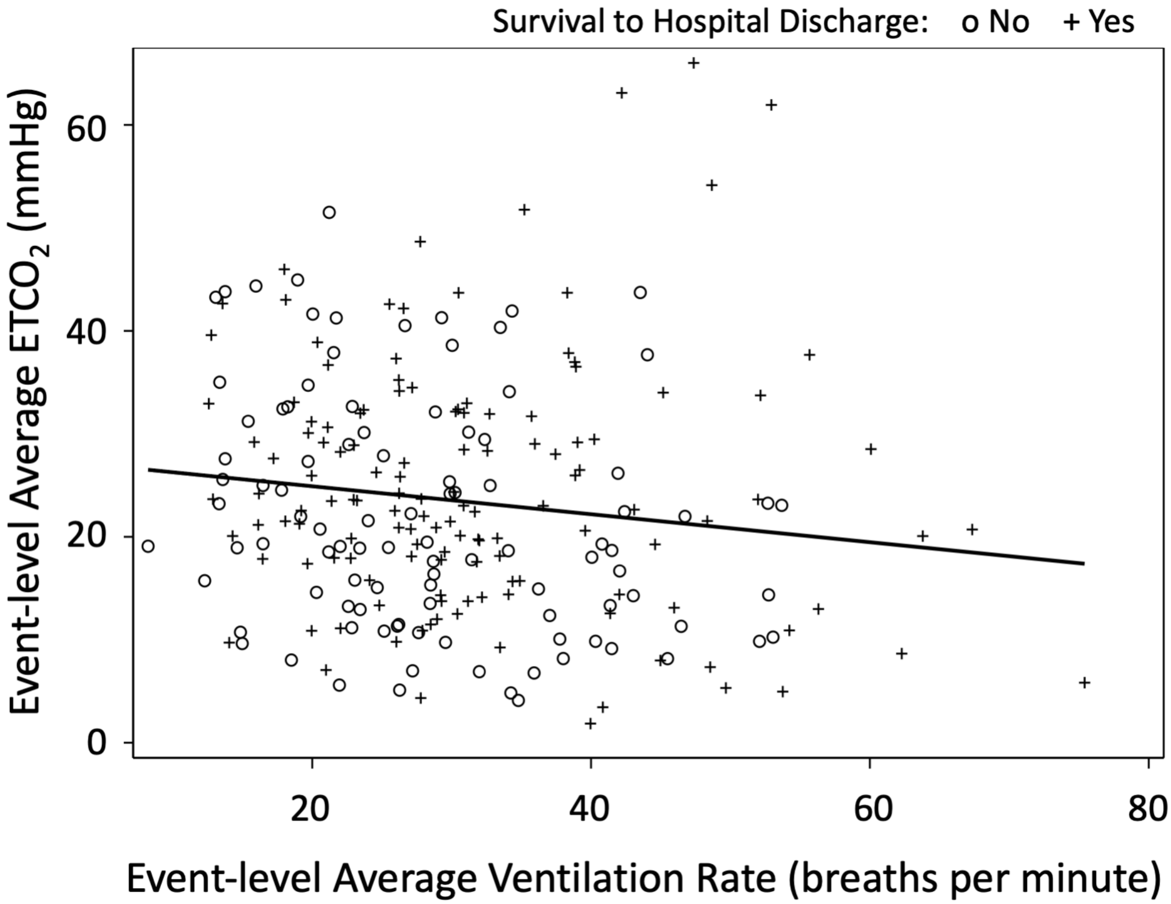

Figure S3: Scatterplot of ETCO2 vs. Ventilation Rate. Each marker represents the event-level average ETCO2 and ventilation rate. The line represents the least squares regression line (regression equation: event-level average ETCO2 = 27.6 - 0.1 * event-level average ventilation rate, p = 0.040).

Appendix: The ICU-RESUS Investigators and the *Eunice Kennedy Shriver* National Institute of Child Health and Human Development Collaborative Pediatric Critical Care Research Network Investigators: Tageldin Ahmed (Department of Pediatrics, Children's Hospital of Michigan, Central Michigan University, Detroit, Michigan), Michael J. Bell (Department of Pediatrics, Children's National Hospital, George Washington University School of Medicine, Washington, DC), Robert Bishop (Department of Pediatrics, University of Colorado School of Medicine and Children's Hospital Colorado, Aurora, Colorado), Matthew Bochkoris (Department of Critical Care Medicine, UPMC Children's Hospital of Pittsburgh, University of Pittsburgh, Pennsylvania), Candice Burns (Department of Pediatrics and Human Development, Michigan State University, Grand Rapids, Michigan), Todd C. Carpenter (Department of Pediatrics, University of Colorado School of Medicine and Children's Hospital Colorado, Aurora, Colorado), Joseph A. Carcillo (Department of Critical Care Medicine, UPMC Children's Hospital of Pittsburgh, University of Pittsburgh, Pittsburgh, Pennsylvania), J. Michael Dean Department of Pediatrics, University of Utah, Salt Lake City, Utah), J. Wesley Diddle (Department of Pediatrics, Children's National Hospital, George Washington University School of Medicine, Washington, DC), Myke Federman (Department of Pediatrics, Mattel Children's Hospital, University of California Los Angeles, Los Angeles, California), Richard Fernandez (Department of Pediatrics, Nationwide Children's Hospital, The Ohio State University, Columbus, Ohio), Ericka L Fink (Department of Critical Care Medicine, UPMC Children's Hospital of Pittsburgh, University of Pittsburgh, Pittsburgh, Pennsylvania), Deborah Franzon (Department of Pediatrics, Benioff Children's Hospital, University of California, San Francisco, San Francisco, California), Aisha H. Frazier (Department of Pediatrics, Nemours/Alfred I. duPont Hospital for Children and Thomas Jefferson University, Wilmington, Delaware), Mark Hall (Department of Pediatrics, Nationwide Children's Hospital, The Ohio State University, Columbus, Ohio), David A. Hehir (Department of Anesthesiology and Critical Care Medicine, Children's Hospital of Philadelphia, University of Pennsylvania, Philadelphia, Pennsylvania), Christopher M. Horvat (Department of Critical Care Medicine, UPMC Children's Hospital of Pittsburgh, University of Pittsburgh, Pittsburgh, Pennsylvania), Leanna L. Huard (Department of Pediatrics, Mattel Children's Hospital, University of California Los Angeles, Los Angeles, California), Tensing Maa (Department of Pediatrics, Nationwide Children's Hospital, The Ohio State University, Columbus, Ohio), Arushi Manga (Department of Pediatrics, Washington University School of Medicine, St. Louis, Missouri), Patrick S. McQuillen (Department of Pediatrics, Benioff Children's Hospital, University of California - San Francisco, San Francisco, California), Maryam Y. Naim (Department of Anesthesiology and Critical Care Medicine, The Children's Hospital of Philadelphia, Philadelphia, Pennsylvania), Daniel Notterman Department of Molecular Biology, Princeton University, Princeton, New Jersey), Murray M. Pollack (Department of Pediatrics, Children's National Hospital, George Washington University School of Medicine, Washington, DC), Anil Sapru (Department of Pediatrics, Mattel Children's Hospital, University of California Los Angeles, Los Angeles, California), Carleen Schneiter (Department of Pediatrics, University of Colorado School of Medicine and Children's Hospital Colorado, Aurora, Colorado), Matthew P. Sharron (Department of Pediatrics, Children's National Hospital, George Washington University School of Medicine, Washington, DC), Sarah Tabbutt (Department of Pediatrics, Benioff Children's Hospital, University of California - San Francisco, San Francisco, California), Shirley Viteri (Department of Pediatrics, Nemours/Alfred I. duPont Hospital for Children and Thomas Jefferson University, Wilmington, Delaware), David Wessel (Department of Pediatrics, Children's National Hospital, George Washington University School of Medicine, Washington, DC), Athena F. Zuppa (Department of Anesthesiology and Critical Care Medicine, Children's Hospital of Philadelphia, University of Pennsylvania, Philadelphia, Pennsylvania).
